# Male infertility risk and gut microbiota: a Mendelian randomization study

**DOI:** 10.3389/fmicb.2023.1228693

**Published:** 2023-09-26

**Authors:** Zhi-da Fu, Yao Wang, Hong-li Yan

**Affiliations:** Center for Reproductive Medicine, Changhai Hospital, Naval Medical University, Shanghai, China

**Keywords:** male infertility, Mendelian randomization, gut microbiota, GWAS, sperm quality

## Abstract

**Background:**

In recent decades, the decline of male sperm quality has become a worldwide phenomenon, with sperm quality of critical importance for the ability to conceive naturally. Recent studies suggest that male fertility function is closely linked to the gut microbiota, however, the cause-and-effect association between the gut microbiota and male infertility risk is currently unclear.

**Methods:**

We performed one two-sample Mendelian randomization (MR) study, which uses summary data on human gut microbiota from the MiBioGen consortium as factors of exposure. FinnGen Consortium R8 data was used to obtain GWAS data for male infertility. To evaluate cause-and-effect associations linking gut microbiota and male infertility risk with multiple Mendelian randomization methods, we included inverse variance weighted (IVW), MR-Egger, and Maximum Likelihood (ML) Ratio. The heterogeneity of instrumental variables was evaluated through Cochran's Q, Rucker's Q, and leave-one-out analysis methods.

**Results:**

We found a positive association between Allisonella, Anaerotruncus, Barnesiella, Intestinibacter, and Lactococcus with male infertility risk according to the MR analysis results. Bacteroides Romboutsia, Ruminococcaceae (NK4A2140group), and Ruminococcaceae (UCG011) play a protective function in male infertility pathogenesis.

**Conclusion:**

It was found that gut microbiota and infertility are causally related in this study. In subsequent studies, there is a need to build a larger and more comprehensive GWAS database on male infertility, which will reveal the underlying mechanisms for gut microbiota and male infertility. There is a need for randomized controlled trials for validating the protective effect of the associated gut microbiota against male infertility risk, and for exploring the associated mechanisms.

## Introduction

As a worldwide human health problem, infertility is widely reported to impact over 186 million individuals globally (Inhorn and Patrizio, 2015). There are numerous theories about the etiology of infertility, however, the specific mechanisms are not yet known. Up to 50% of infertility is due to male factors (Minhas et al., 2021). In recent decades, in both developed and developing countries, male fertility rates have declined, raising concerns among academics and society at large (Gunes et al., 2018). To our knowledge, there are no known molecular biomarkers for male infertility. An important risk factor for male infertility is poor sperm quality. In addition to overweight (Salas-Huetos et al., 2021), substance abuse (Gundersen et al., 2015), and testicular inflammation (Bryan et al., 2020), poor lifestyle habits such as smoking (Sharma et al., 2016), alcohol consumption (Li et al., 2022), and consumption of sugary beverages (Nassan et al., 2021) can affect sperm quality.

The gut microbiota, often referred to as the second human-genome, as a vast bacterial community, is essential for maintaining balance between the external and internal environments of the host. Gut microbiota has been linked to many diseases, including psychiatric disorders (Mangiola et al., 2016), hematological disorders (Yang et al., 2022), and orthopedic disorders (Liu et al., 2019). Studies suggest that there may be a correlation between gut microbiota and male fertility (Zhao et al., 2021). Confounding factors, such as environment, lifestyle, and dietary habits, often influence traditional observational studies and greatly interfere with the inference concerning the relationship of causality of risk factors and endpoint outcomes. In Mendelian randomization (MR), single nucleotide polymorphisms (SNPs), which are instrumental variables (IVs), are used to infer cause-and-effect correlations between exposure factors and outcomes (Greenland, 2000; Bowden and Holmes, 2019). In order to understand the causal relationship between gut microbiota and disease, MR methods are extensively used, and there are no studies on the causality between gut microbiota and male infertility risk. In this study, we will evaluate the existence of cause-and-effect correlation between the gut microbiota and male infertility through the use of two-sample MR.

## Methods

### Study design

For our GWAS data summary of gut microbiota and male infertility based on the Genome-Wide Association Study (GWAS), after screening the IVs that met the screening criteria, we performed MR analysis, and made an inference about the cause-and-effect correlation between the gut microbiota and male infertility. This study was based on the three major hypotheses of MR analysis: (1) the correlation between IVs and exposure will be significant. In this study, the power of IVs was evaluated through the use of the formula, F = R^2^ × (N-k-1)/(k×(1-R^2^)), in which N is the number of exposed samples, k is the number of included SNPs, and R^2^ represents the genetic variable to explain the variance of exposure. When the SNP corresponds to the F-statistic<10, a weak IV is considered, which would cause bias to the results (Xiang et al., 2021); (2) no association exists on IVs and confounding factors which affect the exposure and the outcome; and (3) there is an influence of IVs on outcome by exposure only. For this study, all the datasets used were made public.

### Data sources

MiBioGen consortium (www.mibiogen.org) provides GWAS summary data for the gut microbiota, and it is currently the biggest published source of human-related gut microbiota GWAS data (Kurilshikov et al., 2021). There were 24 cohorts comprising 18,340 participants in the study, including participants from the United States, Canada, Denmark, and the United Kingdom, with the majority of individuals from Europe (*n* = 14,306). This GWAS data includes 211 groups of bacteria, of which there are five types, namely class, phylum, order, family, and genus. The smallest level of the genus was taken as the object in this study. A total of 131 genera have been identified, and 12 of the unknown bacterial genera were excluded, finally incorporating 119 bacterial genera for MR analysis. The GWAS data for male infertility were taken from FinnGen Consortium version R8 (https://r8.finngen.fi/) (FinnGen, 2022; Kurki et al., 2022). The phenotype “male infertility” was used in the study, and this data included 1,128 cases and 110,070 controls.

### Selection of instrumental variables

For ensuring the truthfulness and reliability of causal inference findings from our study, we performed a series of strict qualitative controls. (1) Significantly associated SNPs were selected to potentially be IVs by the threshold of *p* < 1 × 10^−5^ for each genus. (2) 1,000 Genome data Project European samples were used to remove linkage disequilibrium (LD) between IVs, as a strong LD would lead to bias (clumping_distance = 10,000kb and clumping_r^2^ < 0.001). (3) When a palindromic SNP was present, the palindromic SNP was removed. (4) Proxy SNPs significantly associated with IVs by LDlinkR were found when the GWAS outcome data did not exist for SNPs associated with the gut microbiota (r^2^ > 0.8) (Myers et al., 2020).

### Statistical analysis

The study used a variety of MR methods to evaluate and verify the cause-and-effect correlation between gut microbiota and risk of male infertility, including inverse variance weighted (IVW) (Burgess et al., 2013), MR-Egger regression (Bowden et al., 2015), weighted median (Bowden et al., 2016), maximum likelihood (ML) ratio (Pierce and Burgess, 2013), constrained maximum likelihood, model averaging, and Bayesian information criterion (cML-MA-BIC) (Xue et al., 2021). The nature of IVW is a method of meta-analysis (Burgess et al., 2013), which were analyzed by a weighted linear regression to get the overall estimation of the effects of gut microbiota and male infertility. IVW is useful for causal evaluation when no horizontal pleiotropy exists between SNPs. The MR-Egger method shows the horizontal pleiotropy of IVs by using intercept terms if there is pleiotropy in the IVs (Bowden et al., 2015). The results of the MR-Egger regressions and IVW are the same if the intercept term is equal to 0. With up to fifty percent of invalid IVs, a weighted median model can obtain consistent causal estimation relationship (Bowden et al., 2016). ML results have smaller errors than IVW when there is no horizontal pleiotropy and heterogeneity between IVs (Pierce and Burgess, 2013). Based on constrained maximum likelihood with model average, cML-MA-BIC is a method for MR that provides a robust representation of gene pleiotropy effect between IVs (Xue et al., 2021).

SNPs that were included in the analysis for potential horizontal pleiotropy were evaluated by MR-Egger regression (Bowden et al., 2015). Moreover, the pleiotropy and outlier status test was performed on the SNPs included in the analysis; we used the MR-PRESSO method (Verbanck et al., 2018). To evaluate the heterogeneity of the MR analysis, the Cochran's Q statistic (IVW) and Rucker's Q statistic (MR-Egger) were used, with *p* > 0.05 meaning no heterogeneity (Yang et al., 2023). In order to determine the existence of potentially strong influential instrumental variables, each instrumental variable was excluded sequentially by using leave-one-out sensitivity analysis. Study with R software (version 4.2.1), TwoSampleMR package (version 0.5.6) (Hemani et al., 2017), LDlinkR (version 1.2.3) (Myers et al., 2020), and MR-PRESSO (version 1.0) (Verbanck et al., 2018) was used for MR analysis.

## Results

### Instrumental variables selection

Following the selection by a threshold of *p* < 1 × 10^−5^, the SNPs significantly associated with 119 gut microbiota genera were selected and SNPs with a significant LD influence were removed, leaving 1,269 remaining SNPs that were used as the IVs for further analysis. This is detailed in Supplementary Table 1, and all the available IVs details are displayed in Supplementary Table 2.

### MR analysis

In total, 10 bacterial genera with a causal relationship with male infertility shown through at least one MR method were found (*p* < 0.05): Allisonella, Anaerofilum, Anaerotruncus, Bacteroides, Barnesiella, Intestinibacter, Lactococcus, Romboutsia, Ruminococcaceae (NK4A214group), and Ruminococcaceae (UCG011) (Table 1).

**Table 1:** MR results of causal links between gut microbiota and male infertility (*p* < 1 × 10^−5^).

**bacterial taxa (exposure)**	**Nsnp**	**Methods**	**Beta**	**SE**	**P-value**	**OR (CI)**	**F statistic**
Allisonella	8	IVW	0.25	0.15	0.091	1.28 (0.96–1.71)	23.75
Allisonella	8	MR-Egger	1.07	1.02	0.333	2.92 (0.4–21.46)	
Allisonella	8	WM	0.21	0.18	0.241	1.24 (0.87–1.78)	
Allisonella	8	ML	0.26	0.13	0.045	1.3 (1.01–1.68)	
Allisonella	8	cML-MA-BIC	0.24	0.14	0.089	1.27 (0.96–1.67)	
Anaerofilum	10	IVW	−0.35	0.21	0.097	0.7 (0.46–1.07)	23.61
Anaerofilum	10	MR-Egger	−0.8	1.22	0.531	0.45 (0.04–4.93)	
Anaerofilum	10	WM	−0.24	0.21	0.257	0.79 (0.52–1.19)	
Anaerofilum	10	ML	−0.38	0.15	0.012	0.68 (0.5–0.92)	
Anaerofilum	10	cML-MA-BIC	−0.2	0.18	0.285	0.82 (0.57–1.18)	
Anaerotruncus	13	IVW	0.67	0.28	0.016	1.96 (1.13–3.4)	23.4
Anaerotruncus	13	MR-Egger	1.16	0.85	0.196	3.21 (0.61–16.86)	
Anaerotruncus	13	WM	0.23	0.36	0.513	1.26 (0.63–2.54)	
Anaerotruncus	13	ML	0.7	0.24	0.004	2.01 (1.26–3.2)	
Anaerotruncus	13	cML-MA-BIC	0.61	0.29	0.033	1.84 (1.05–3.24)	
Bacteroides	9	IVW	−0.54	0.27	0.048	0.58 (0.34–0.99)	22.74
Bacteroides	9	MR-Egger	−0.6	1.42	0.686	0.55 (0.03–8.88)	
Bacteroides	9	WM	−0.39	0.35	0.263	0.68 (0.34–1.34)	
Bacteroides	9	ML	−0.54	0.28	0.052	0.58 (0.34–1.01)	
Bacteroides	9	cML-MA-BIC	−0.53	0.28	0.062	0.59 (0.34–1.03)	
Barnesiella	14	IVW	0.34	0.25	0.175	1.41 (0.86–2.31)	22.93
Barnesiella	14	MR-Egger	−0.17	0.99	0.863	0.84 (0.12–5.8)	
Barnesiella	14	WM	0.53	0.3	0.072	1.7 (0.95–3.03)	
Barnesiella	14	ML	0.35	0.21	0.092	1.42 (0.94–2.15)	
Barnesiella	14	cML-MA-BIC	0.56	0.23	0.017	1.75 (1.11–2.76)	
Intestinibacter	15	IVW	0.42	0.23	0.074	1.51 (0.96–2.39)	22.78
Intestinibacter	15	MR-Egger	0.64	0.78	0.429	1.89 (0.41–8.79)	
Intestinibacter	15	WM	0.27	0.27	0.32	1.31 (0.77–2.23)	
Intestinibacter	15	ML	0.43	0.19	0.021	1.53 (1.07–2.2)	
Intestinibacter	15	cML-MA-BIC	0.32	0.22	0.154	1.37 (0.89–2.12)	
Lactococcus	9	IVW	0.25	0.15	0.085	1.29 (0.97–1.72)	22.83
Lactococcus	9	MR-Egger	−0.14	0.69	0.841	0.87 (0.22–3.36)	
Lactococcus	9	WM	0.41	0.2	0.039	1.51 (1.02–2.22)	
Lactococcus	9	ML	0.26	0.15	0.071	1.3 (0.98–1.73)	
Lactococcus	9	cML-MA-BIC	0.25	0.15	0.098	1.28 (0.95–1.73)	
Romboutsia	13	IVW	−0.41	0.22	0.067	0.67 (0.43–1.03)	29.1
Romboutsia	13	MR-Egger	−1.35	0.6	0.046	0.26 (0.08–0.84)	
Romboutsia	13	WM	−0.17	0.3	0.571	0.84 (0.47–1.52)	
Romboutsia	13	ML	−0.42	0.21	0.05	0.66 (0.43–1)	
Romboutsia	13	cML-MA-BIC	−0.37	0.23	0.108	0.69 (0.44–1.08)	
Ruminococcaceae (NK4A214group)	13	IVW	−0.57	0.23	0.014	0.56 (0.36–0.89)	22.68
Ruminococcaceae (NK4A214group)	13	MR-Egger	−1.08	0.78	0.191	0.34 (0.07–1.55)	
Ruminococcaceae (NK4A214group)	13	WM	−0.19	0.32	0.545	0.83 (0.44–1.53)	
Ruminococcaceae (NK4A214group)	13	ML	−0.59	0.22	0.007	0.55 (0.36–0.85)	
Ruminococcaceae (NK4A214group)	13	cML-MA-BIC	−0.51	0.26	0.047	0.6 (0.36–0.99)	
Ruminococcaceae (UCG011)	8	IVW	−0.27	0.13	0.042	0.76 (0.59–0.99)	23.72
Ruminococcaceae (UCG011)	8	MR-Egger	−0.56	0.66	0.429	0.57 (0.16–2.08)	
Ruminococcaceae (UCG011)	8	WM	−0.23	0.17	0.181	0.8 (0.57–1.11)	
Ruminococcaceae (UCG011)	8	ML	−0.27	0.13	0.045	0.76 (0.59–0.99)	
Ruminococcaceae (UCG011)	8	cML-MA-BIC	−0.27	0.14	0.048	0.77 (0.59–1)	

IVW analysis showed that Anaerotruncus (odds ratio (OR) = 1.96, 95% confidence interval (CI), 1.13–3.38, *p* = 0.016) was positively associated with male infertility risk. Bacteroides (OR = 0.58, 95% CI, 0.34- 0.99, *p* = 0.048), Ruminococcaceae (NK4A214group) (OR = 0.57, 95% CI, 0.36–0.89, *p* = 0.014), and Ruminococcaceae (UCG011) (OR = 0.76, 95% CI, 0.59–0.99, *p* = 0.042) were negatively associated with male infertility risk. MR Egger's MR estimates showed that Romboutsia (OR = 0.26, 95% CI, 0.08–0.84, *p* = 0.046) was shown to be negatively correlated with male infertility risk. Risk of male infertility was shown to be positively associated with Lactococcus (OR = 1.51, 95% CI, 1.02–2.23, *p* = 0.045) by weighted median MR estimates. Maximum likelihood ratio MR estimates showed that Allisonella (OR = 1.3, 95% CI, 1.01–1.68, *p* = 0.045), Anaerotruncus (OR = 2.01, 95% CI, 1.26–3.2, *p* = 0.004), and Intestinibacter (OR = 1.53, 95% CI, 1.07–2.2, *p* = 0.021) had a positive relationship with male infertility risk. Anaerofilum (OR = 0.68, 95% CI, 0.5–0.92, *p* = 0.012), Ruminococcaceae (NK4A214group) (OR = 0.55, 95% CI, 0.36–0.85, *p* = 0.007), and Ruminococcaceae (UCG011) (OR = 0.76, 95% CI, 0.59–0.99, *p* = 0.045) were protective against the development of infertility in men. The cML-MA-BIC results showed that Anaerotruncus (OR = 1.84, 95% CI, 1.05–3.24, *p* = 0.033) and Barnesiella (OR = 1.75, 95% CI, 1.11–2.76, *p* = 0.017) were correlated with a positive effect of male infertility risk, and Ruminococcaceae (NK4A214group) (OR = 0.6, 95% CI, 0.36–0.99, *p* = 0.047) and Ruminococcaceae (UCG011) (OR = 0.77, 95% CI, 0.59–0.99, *p* = 0.048) were protective against the development of male infertility.

**Figure 1:**
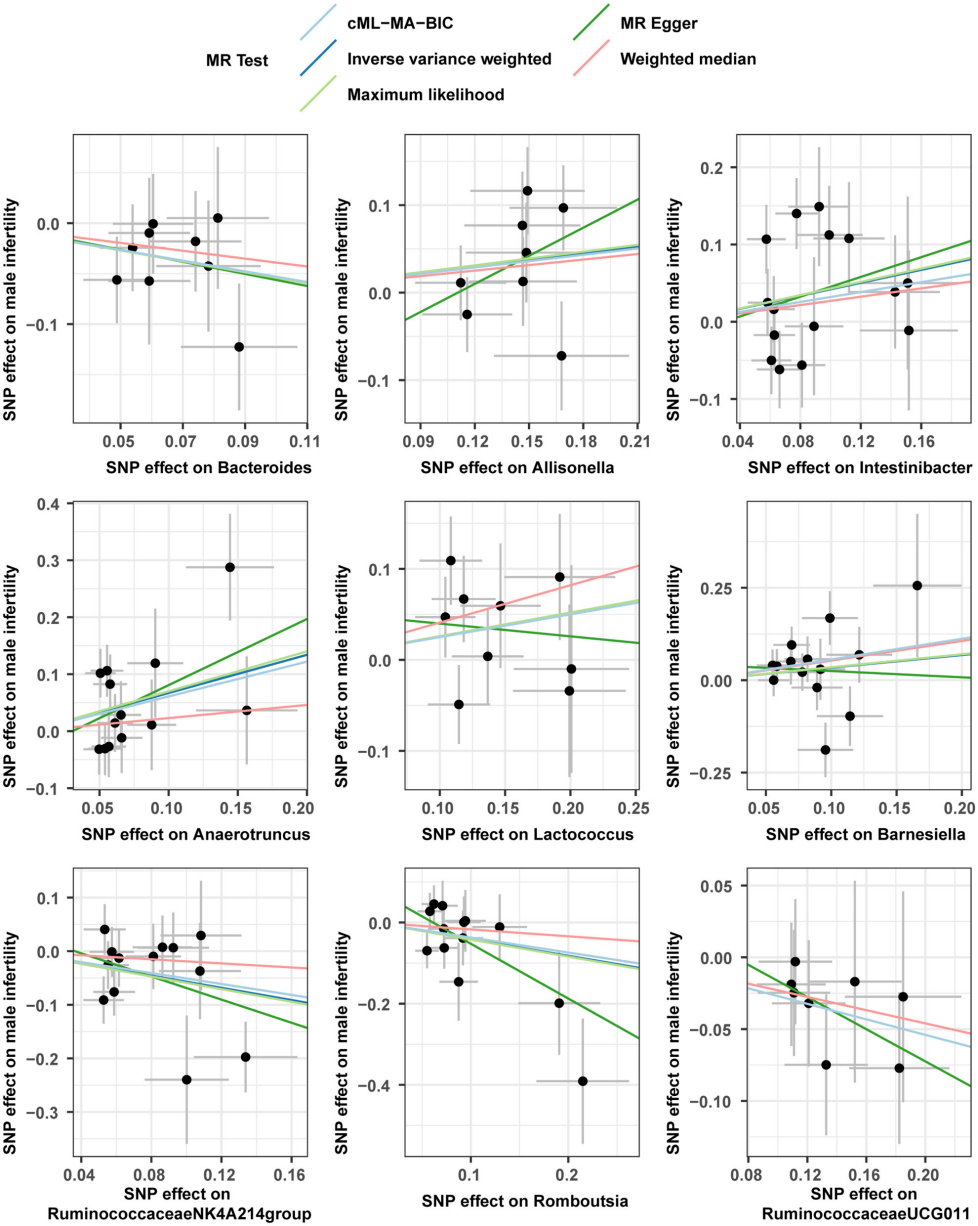
Scatter plots for the casual association between gut microbiota and male infertility.

**Figure 2:**
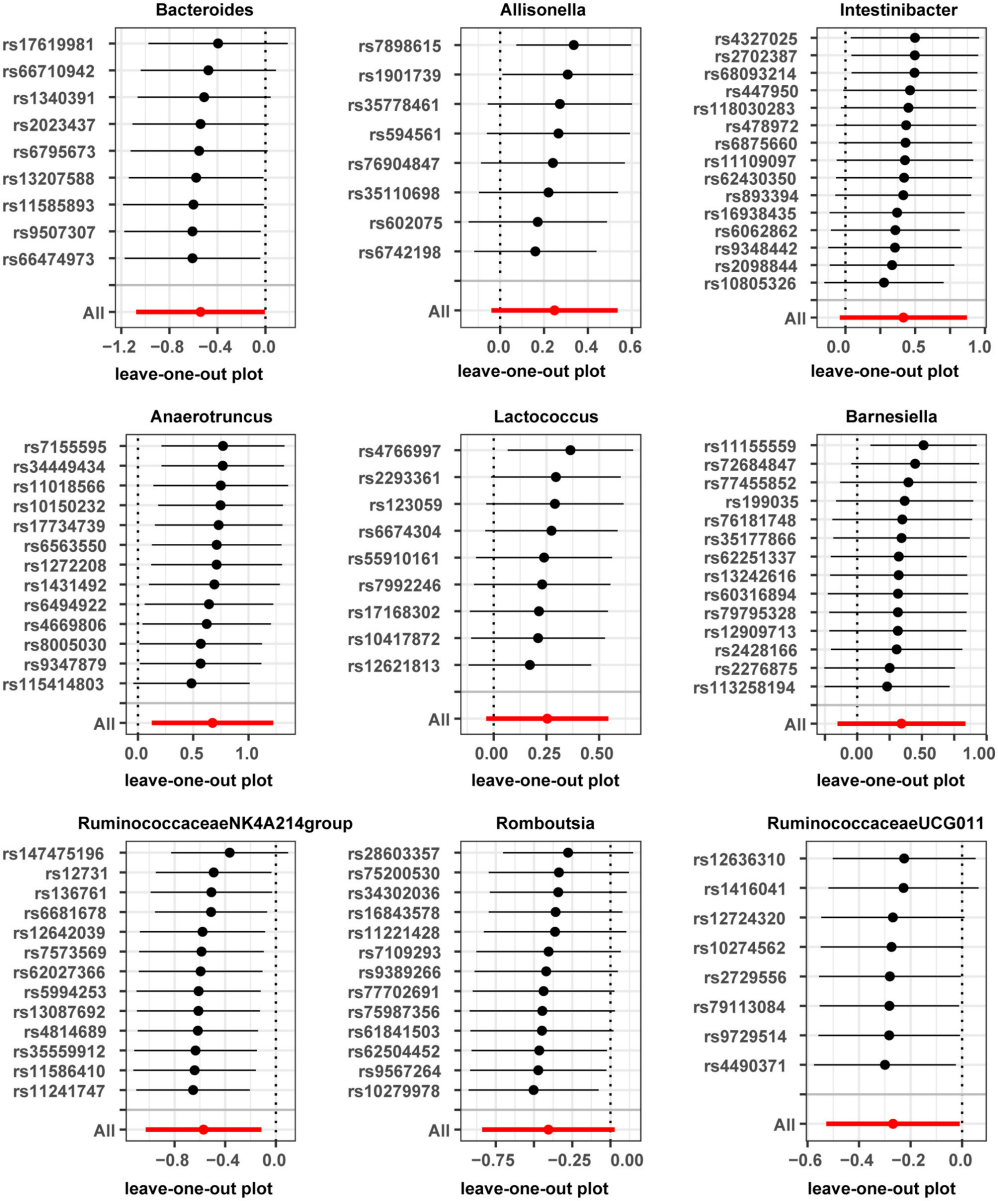
Leave-one-out plots for casual association between gut microbiota and male infertility.

Horizontal pleiotropy was evaluated between instrumental variables by MR-Egger regression, and results are shown in Supplementary Table 3, with no horizontal pleiotropy found for gut microbiota. A global test of MRPRESSO, detailed in Supplementary Table 4, shows that MR analysis between Anaerofilum and male infertility has a horizontal pleiotropy (*p* < 0.05), and a significant outlier rs4506496 for Anaerofilum was found by the aberration test of MR-PRESSO analysis. After removing this outlier, MR analysis was performed again, as shown in Supplementary Table 5, and showed that no significant cause-and-effect correlation was found between Anaerofilum and male infertility (*p* > 0.05). Quantitative analysis of the 10 bacterial genera was carried out using Cochran's Q and Rucker's Q, respectively, and the overview of the results is shown in Supplementary Table 6. Heterogeneity (*p* < 0.05) in MR analysis between Anaerofilum and Intestinibacter and male infertility is shown in Supplementary Table 6. MR analysis for bacterial genera with heterogeneity was performed again with a random effects model. Calibrated IVW results showed Intestinibacter (OR = 1.51, 95% CI, 1.07–2.15, *p* = 0.020) and Anaerofilum (OR = 0.81, 95% CI, 0.60–1.10, *p* = 0.181) had an effect on male fertility, which is in agreement with the results from the ML method and MRPRESSO analysis, respectively. Allisonella, Bacteroides, Barnesiella, Intestinibacter, Lactococcus, and Romboutsia showed potential outliers in scatter plots (Figure 1) and leave-one-out analysis plots (Figure 2), however, no significant outliers were found by MRPRESSO analysis. The forest plot and funnel plot are displayed in our Supplementary Figure.

Thus, in our study we showed that Allisonella, Anaerotruncus, Barnesiella, Intestinibacter, and Lactococcus were positively associated with the risk of male infertility. Bacteroides, Romboutsia, Ruminococcaceae (NK4A2140group), and Ruminococcaceae (UCG011) played a protective role in the pathogenesis of male infertility.

## Discussion

Infertility, which is a worldwide public health problem (Inhorn and Patrizio, 2015), has been closely associated with the reduction of newborns and increased population aging, and could have a serious potential negative influence on social development. Throughout the human digestive tract, mucosal surfaces are colonized by a host of symbiotic microbes. Gut microbiota affect host systems such as digestion (Franzosa et al., 2019), immunity (Thaiss et al., 2016), and the nervous system (Sharon et al., 2016). In our current study, we aimed to discover whether male infertility is influenced by gut microbiota. It is the first two-sample Mendelian randomization study to examine the cause-and-effect links between gut microbiota and male infertility risk using the European cohort, to our knowledge.

Through the use of the MR method, 119 gut microbiota and male infertility were causally studied at the genetic level, effectively avoiding the confounding variables that can interfere with an observational study. In our study, we found a positive and direct genetic causality between human gut microbiota and male infertility, with Allisonella, Anaerotruncus, Barnesiella, Intestinibacter, and Lactococcus all showing to have an effect. Furthermore, Bacteroides Romboutsia, Ruminococcaceae (NK4A214group), and Ruminococcaceae (UCG011) were found to prevent male infertility. Combined with our study and a pubmed search, Allisonella and Intestinibacter were not reported to be associated with male infertility, which provided a new possible way to investigate male infertility and contributes positively to the genetic study of male infertility.

Several observational studies reported the association of gut microbiota and reproductive capacity in mammals. A potential mechanism for alteration of the reproductive system in male rats was found through the disruption of the gut microbiota by Ditridecyl-phthalate (Zhao et al., 2020a). Gut microbiota could also affect testicular secretion function of mice (Al-Asmakh et al., 2014). Translocation of the gut microbiota, through a multiple immunological mechanism, causes inflammation of the testicles and epididymis, which affects spermatogenesis (Wang and Xie, 2022). Self-reported stress was negatively associated with semen quality in a cross-sectional study (Nordkap et al., 2016). Gut flora is closely associated with the hypothalamic-pituitary-adrenal (HPA) axis, and the HPA regulates a series of physical processes to respond to external stresses (Foster and McVey Neufeld, 2013; Farzi et al., 2018). One animal experiment has reported the increased abundance of Anaerotruncus in prenatal stress (PNS) animal models (Golubeva et al., 2015), which suggests Anaerotruncus as a possible biomarker of stress. It has been shown that diabetes has negative effects on male fertility (Chen et al., 2021; Lotti and Maggi, 2023). The abundance of Anaerotruncus was significantly increased in mice that had testicular injury caused by diabetes, however, its abundance was significantly reduced after treatment with cornuside (Cor), which suggested that Anaerotruncus could be a potential biomarker of testicular injury (Liu et al., 2021); this is in line with our findings. Vitamin A has a critical effect on the process of spermatogenesis (Hogarth and Griswold, 2010). Mice with metabolic syndrome (Mets) have abnormal testicular spermatogenesis due to impaired vitamin A absorption in the intestine, which may be associated with the significantly reduced abundance of Ruminococcaceae (NK4A214group) (Zhang et al., 2022). As an androgen found in epididymal cells, dihydrotestosterone (DHT) regulates sperm maturation (Robaire and Henderson, 2006), and a large-scale animal study suggested that an abundance of Ruminococcaceae was positively correlated with DHT (Chen et al., 2023). Regulation of the gut microbiota to improve the symptoms of drug-induced male infertility was demonstrated by several studies (Zhao et al., 2020b; Sun et al., 2022). Triptolide -induced testicular injury mouse model results in an increase in relative abundance of Firmicutes and a decrease in relative abundance of Parabacteroides, causing the reduction of spermine synthesis. By transferring normal microbiota into mice, testicular dysfunction can be reversed (Zhao et al., 2021). Busulfan chemotherapy is an effective treatment for leukemia (Allan, 1989), although a side effect of this is male infertility. In the intestinal tract, Busulfan will increase the abundance of Firmicutes and Proteobacteria (Zhao et al., 2020b). Chestnut polysaccharides can restore the imbalance of gut microbiota and thus restore the damaged spermatogenesis caused by Busulfan (Sun et al., 2022). Alginate oligosaccharides (AOS) improve male infertility after Busulfan application with the improvement of gut microbiota (Zhao et al., 2020b). Research has demonstrated that AOS may improve sperm quality by increasing Bacteroides' abundance and mitigating Mucispirillum abundance in the gut microbiota (Hao et al., 2022).

A Westernized diet (a diet high in high saturated fatty acids and refined sugar refined) has been reported as a significant factor in the long-term decline in human sperm concentrations (Wu et al., 2011). Research indicates that the different types of diet patterns based on saturated, monounsaturated, or polyunsaturated fatty acids may induce alterations in gut microbiota composition (Patterson et al., 2014). The fatty acids level (Bunay et al., 2021), especially DHA, are associated with spermatogenesis (Hale et al., 2019). A high-fat diet (HFD) induces disruption of the gut microbiome, as reflected by decreases in Bacteroides and increases in Firmicutes and Aspergillus in mice models (Hildebrandt et al., 2009; Graham et al., 2015). Moreover, HFD also leads to alterations in the mucosal epithelium of the intestine, which results in intestinal barrier dysfunction (Moreira et al., 2012), higher permeability (Ji et al., 2011), and more diffusion of endotoxins into the bloodstream (Ji et al., 2011). In mice, excess endotoxin activates the immune system, resulting in an increase in proinflammatory factors in the epididymis, and decreasing sperm viability (Ding et al., 2020).

The major metabolism products of the gut microbiota are short-chain fatty acids (SCFA), which are essential for intestinal immunity and health of the host (Martin-Gallausiaux et al., 2021). Research shows that butyric acid may have a facilitative effect on spermatogenesis (Yan et al., 2022). For our study, the bacteria Ruminococcaceae (NK4A214group) (Verhoeven et al., 2021), Ruminococcaceae (UCG011) (Radjabzadeh et al., 2022), and Romboutsia (Qin et al., 2021) were able to produce butyric acid. Major sources of gastrointestinal epithelium are SCFAs (Martin-Gallausiaux et al., 2021), which are closely associated with maintaining the mucosal barrier function of the intestinal tract. An animal study reported that the dietary fiber supplements improve spermatogenesis and semen quality by promoting SCFA production through improved gut microbiota (Lin et al., 2022). A metabolically healthier gut microbiota may be achieved by a diet that is supplemented with low animal fat, low animal protein, and high fiber (Fan and Pedersen, 2021), to prevent endotoxin diffusion into blood; this might improve infertility in males in some cases. However, this requires a further randomized controlled experiment to demonstrate.

Our study contains several strengths. (1) The data on gut microbiota and male infertility were obtained from a European population, effectively avoiding bias caused by differences in ethnicity; this can better reflect the cause-and-effect correlation between gut microbiota and male infertility. (2) Gut microbiota inherited variants from the largest usable GWAS metadata, which guarantees power of the IVs for the analysis of MR. (3) Genetic variants found in gut microbiota were derived from the maximum amount of GWAS metadata available, ensuring IV power in MR analyses. (4) Our study met all three assumptions of the MR analysis. To elaborate, we screened SNPs that were closely related with the gut microbiota (*p* < 1 × 10^−5^) and passed the F-statistic checks (F > 10), which satisfied the first assumption. The screened SNPs were evaluated by LD analysis, which fulfilled the second assumption. We conducted a study in which we used MR-Egger regression and MRPRESSO analyses to exclude SNPs with horizontal pleiotropy, and the last acquired SNPs satisfied the third hypothesis. (5) There may be some reduction in interference from confounding factors and reverse causation with MR methods. However, it is also important to note that our study has some limitations. Firstly, the results of the analysis, which was performed only for European populations, were not sufficiently well representative of populations in other regions. Secondly, there may be a potential bias in the study as the number of cases in the GWAS data for male infertility is relatively small. And finally, GWAS of male infertility were not categorized, so our study was not well able to reveal the association of gut microbiota with specific types of male infertility. Future studies and randomized controlled trials are required to better reveal the cause-and-effect correlation between gut microbiota and male infertility.

## Conclusion

In summary, the MR analysis performed using data of a large sample of GWAS analysis showed a cause-and-effect relationship between gut microbiota and male infertility. However, greater GWAS databases need to be established for the exploration of mechanisms between gut microbiota and male infertility. There are several gut bacteria identified that may reduce the prevalence of male infertility and could offer hope in treating and preventing male infertility.

## Data availability statement

The datasets presented in this study can be found in online repositories. The names of the repository/repositories and accession number(s) can be found in the article/Supplementary material.

## Ethics statement

Ethical approval was not required for the study involving humans in accordance with the local legislation and institutional requirements. Written informed consent to participate in this study was not required from the participants or the participants' legal guardians/next of kin in accordance with the national legislation and the institutional requirements.

## Author contributions

Conceptualization and writing—original draft preparation: Z-dF and YW. Writing—review and editing: H-lY. All authors contributed to the article and approved the submitted version.

## References

[B1] Al-Asmakh M. Stukenborg J. B. Reda A. Anuar F. Strand M. L. Hedin L.. (2014). The gut microbiota and developmental programming of the testis in mice. PLoS ONE 9, e103809. 10.1371/journal.pone.0103809 25118984 PMC4132106

[B2] Allan N. C. (1989). Therapeutic options in chronic myeloid leukaemia. Blood Rev. 3, 45–52. 10.1016/0268-960X(89)90024-6 2650775

[B3] Bowden J. Davey Smith G. Burgess S. (2015). Mendelian randomization with invalid instruments: effect estimation and bias detection through Egger regression. Int. J. Epidemiol. 44, 512–525. 10.1093/ije/dyv080 26050253 PMC4469799

[B4] Bowden J. Davey Smith G. Haycock P. C. Burgess S. (2016). Consistent estimation in mendelian randomization with some invalid instruments using a weighted median estimator. Genet. Epidemiol. 40, 304–314. 10.1002/gepi.21965 27061298 PMC4849733

[B5] Bowden J. Holmes M. V. (2019). Meta-analysis and Mendelian randomization: a review. Res. Synth. Methods 10, 486–496. 10.1002/jrsm.1346 30861319 PMC6973275

[B6] Bryan E. R. Redgrove K. A. Mooney A. R. Mihalas B. P. Sutherland J. M. Carey A. J.. (2020). Chronic testicular chlamydia muridarum infection impairs mouse fertility and offspring developmentdagger. Biol. Reprod. 102, 888–901. 10.1093/biolre/ioz229 31965142 PMC7124966

[B7] Bunay J. Gallardo L. M. Torres-Fuentes J. L. Aguirre-Arias M. V. Orellana R. Sepulveda N.. (2021). A decrease of docosahexaenoic acid in testes of mice fed a high-fat diet is associated with impaired sperm acrosome reaction and fertility. Asian J. Androl. 23, 306–313. 10.4103/aja.aja_76_20 33269725 PMC8152421

[B8] Burgess S. Butterworth A. Thompson S. G. (2013). Mendelian randomization analysis with multiple genetic variants using summarized data. Genet. Epidemiol. 37, 658–665. 10.1002/gepi.21758 24114802 PMC4377079

[B9] Chen H. Murray E. Sinha A. Laumas A. Li J. Lesman D.. (2021). Dissecting mammalian spermatogenesis using spatial transcriptomics. Cell Rep. 37, 109915. 10.1016/j.celrep.2021.109915 34731600 PMC8606188

[B10] Chen X. Wang Z. Su J. Li H. Xiong J. Fu K.. (2023). Altitude-dependent metabolite biomarkers reveal the mechanism of plateau pika adaptation to high altitudes. Integr. Zool. 2023, 710. 10.1111/1749-4877.12710 36880690

[B11] Ding N. Zhang X. Zhang X. D. Jing J. Liu S. S. Mu Y. P.. (2020). Impairment of spermatogenesis and sperm motility by the high-fat diet-induced dysbiosis of gut microbes. Gut 69, 1608–1619. 10.1136/gutjnl-2019-319127 31900292 PMC7456731

[B12] Fan Y. Pedersen O. (2021). Gut microbiota in human metabolic health and disease. Nat. Rev. Microbiol. 19, 55–71. 10.1038/s41579-020-0433-9 32887946

[B13] Farzi A. Frohlich E. E. Holzer P. (2018). Gut microbiota and the neuroendocrine system. Neurotherapeutics. 15, 5–22. 10.1007/s13311-017-0600-5 29380303 PMC5794709

[B14] FinnGen (2022). FinnGen R8 Release. Available online at: https://r8.finngen.fi/ (accessed December 1, 2022).

[B15] Foster J. A. McVey Neufeld K. A. (2013). Gut-brain axis: how the microbiome influences anxiety and depression. Trends Neurosci. 36, 305–312. 10.1016/j.tins.2013.01.005 23384445

[B16] Franzosa E. A. Sirota-Madi A. Avila-Pacheco J. Fornelos N. Haiser H. J. Reinker S.. (2019). Gut microbiome structure and metabolic activity in inflammatory bowel disease. Nat. Microbiol. 4, 293–305. 10.1038/s41564-018-0306-4 30531976 PMC6342642

[B17] Golubeva A. V. Crampton S. Desbonnet L. Edge D. O'Sullivan O. Lomasney K. W.. (2015). Prenatal stress-induced alterations in major physiological systems correlate with gut microbiota composition in adulthood. Psychoneuroendocrinology 60, 58–74. 10.1016/j.psyneuen.2015.06.002 26135201

[B18] Graham C. Mullen A. Whelan K. (2015). Obesity and the gastrointestinal microbiota: a review of associations and mechanisms. Nutr. Rev. 73, 376–385. 10.1093/nutrit/nuv004 26011912

[B19] Greenland S. (2000). An introduction to instrumental variables for epidemiologists. Int. J. Epidemiol. 29, 722–729. 10.1093/ije/29.4.722 10922351

[B20] Gundersen T. D. Jorgensen N. Andersson A. M. Bang A. K. Nordkap L. Skakkebaek N. E.. (2015). Association between use of marijuana and male reproductive hormones and semen quality: a study among 1,215 healthy young men. Am J Epidemiol. 182, 473–481. 10.1093/aje/kwv135 26283092

[B21] Gunes S. Metin Mahmutoglu A. Arslan M. A. Henkel R. (2018). Smoking-induced genetic and epigenetic alterations in infertile men. Andrologia. 50, e13124. 10.1111/and.13124 30132931

[B22] Hale B. J. Fernandez R. F. Kim S. Q. Diaz V. D. Jackson S. N. Liu L.. (2019). Acyl-CoA synthetase 6 enriches seminiferous tubules with the omega-3 fatty acid docosahexaenoic acid and is required for male fertility in the mouse. J. Biol. Chem. 294, 14394–14405. 10.1074/jbc.RA119.009972 31399511 PMC6768642

[B23] Hao Y. Feng Y. Yan X. Chen L. Ma X. Tang X.. (2022). Gut microbiota-testis axis: FMT mitigates high-fat diet-diminished male fertility via improving systemic and testicular metabolome. Microbiol. Spectr. 10, e0002822. 10.1128/spectrum.00028-22 35446112 PMC9241630

[B24] Hemani G. Tilling K. Davey Smith G. (2017). Orienting the causal relationship between imprecisely measured traits using GWAS summary data. PLoS Genet. 13, e1007081. 10.1371/journal.pgen.1007081 29149188 PMC5711033

[B25] Hildebrandt M. A. Hoffmann C. Sherrill-Mix S. A. Keilbaugh S. A. Hamady M. Chen Y. Y.. (2009). High-fat diet determines the composition of the murine gut microbiome independently of obesity. Gastroenterology. 137, 1716–1724. 10.1053/j.gastro.2009.08.042 19706296 PMC2770164

[B26] Hogarth C. A. Griswold M. D. (2010). The key role of vitamin A in spermatogenesis. J. Clin. Invest. 120, 956–962. 10.1172/JCI41303 20364093 PMC2846058

[B27] Inhorn M. C. Patrizio P. (2015). Infertility around the globe: new thinking on gender, reproductive technologies and global movements in the 21st century. Hum. Reprod. Update 21, 411–426. 10.1093/humupd/dmv016 25801630

[B28] Ji Y. Sakata Y. Tso P. (2011). Nutrient-induced inflammation in the intestine. Curr. Opin. Clin. Nutr. Metab. Care. 14, 315–321. 10.1097/MCO.0b013e3283476e74 21587069 PMC4520304

[B29] Kurilshikov A. Medina-Gomez C. Bacigalupe R. Radjabzadeh D. Wang J. Demirkan A.. (2021). Large-scale association analyses identify host factors influencing human gut microbiome composition. Nat. Genet. 53, 156–165. 10.1038/s41588-020-00763-1 33462485 PMC8515199

[B30] Kurki M. I. Karjalainen J. Palta P. Sipilä T. P. Kristiansson K. Donner K.. (2022). FinnGen: unique genetic insights from combining isolated population and national health register data. medRxiv [Preprint]. 10.1101/2022.03.03.22271360

[B31] Li H. Li N. Lu Q. Yang J. Zhao J. Zhu Q.. (2022). Chronic alcohol-induced dysbiosis of the gut microbiota and gut metabolites impairs sperm quality in mice. Front. Microbiol. 13, 1042923. 10.3389/fmicb.2022.1042923 36532416 PMC9751024

[B32] Lin Y. Wang K. Che L. Fang Z. Xu S. Feng B.. (2022). The improvement of semen quality by dietary fiber intake is positively related with gut microbiota and SCFA in a boar model. Front. Microbiol. 13, 863315. 10.3389/fmicb.2022.863315 35633720 PMC9130837

[B33] Liu L. Shu A. Zhu Y. Chen Y. (2021). Cornuside alleviates diabetes mellitus-induced testicular damage by modulating the gut microbiota. Evid. Compl. Alternat. Med. 2021, 5301942. 10.1155/2021/5301942 34497657 PMC8421159

[B34] Liu Y. Ding W. Wang H. L. Dai L. L. Zong W. H. Wang Y. Z.. (2019). Gut microbiota and obesity-associated osteoarthritis. Osteoarthritis Cartilage 27, 1257–1265. 10.1016/j.joca.2019.05.009 31146016

[B35] Lotti F. Maggi M. (2023). Effects of diabetes mellitus on sperm quality and fertility outcomes: clinical evidence. Andrology. 11, 399–416. 10.1111/andr.13342 36416060

[B36] Mangiola F. Ianiro G. Franceschi F. Fagiuoli S. Gasbarrini G. Gasbarrini A.. (2016). Gut microbiota in autism and mood disorders. World J. Gastroenterol. 22, 361–368. 10.3748/wjg.v22.i1.361 26755882 PMC4698498

[B37] Martin-Gallausiaux C. Marinelli L. Blottiere H. M. Larraufie P. Lapaque N. S. C. F. A. (2021). mechanisms and functional importance in the gut. Proc. Nutr. Soc. 80, 37–49. 10.1017/S0029665120006916 32238208

[B38] Minhas S. Bettocchi C. Boeri L. Capogrosso P. Carvalho J. Cilesiz N. C.. (2021). European association of urology guidelines on male sexual and reproductive health: 2021 update on male infertility. Eur. Urol. 80, 603–620. 10.1016/j.eururo.2021.08.014 34511305

[B39] Moreira A. P. Texeira T. F. Ferreira A. B. Peluzio Mdo C. Alfenas Rde C. (2012). Influence of a high-fat diet on gut microbiota, intestinal permeability and metabolic endotoxaemia. Br. J. Nutr. 108, 801–809. 10.1017/S0007114512001213 22717075

[B40] Myers T. A. Chanock S. J. Machiela M. J. (2020). LDlinkR: an R package for rapidly calculating linkage disequilibrium statistics in diverse populations. Front. Genet. 11, 157. 10.3389/fgene.2020.00157 32180801 PMC7059597

[B41] Nassan F. L. Priskorn L. Salas-Huetos A. Halldorsson T. I. Jensen T. K. Jorgensen N.. (2021). Association between intake of soft drinks and testicular function in young men. Hum. Reprod. 36, 3036–3048. 10.1093/humrep/deab179 34585250 PMC8600659

[B42] Nordkap L. Jensen T. K. Hansen A. M. Lassen T. H. Bang A. K. Joensen U. N.. (2016). Psychological stress and testicular function: a cross-sectional study of 1,215 Danish men. Fertil. Steril. 105, 174–187. 10.1016/j.fertnstert.2015.09.016 26477499

[B43] Patterson E. O'Doherty R. M. Murphy E. F. Wall R. O'Sullivan O. (2014). Impact of dietary fatty acids on metabolic activity and host intestinal microbiota composition in C57BL/6J mice. Br. J. Nutr. 111, 1905–1917. 10.1017/S0007114514000117 24555449

[B44] Pierce B. L. Burgess S. (2013). Efficient design for Mendelian randomization studies: subsample and 2-sample instrumental variable estimators. Am. J. Epidemiol. 178, 1177–1184. 10.1093/aje/kwt084 23863760 PMC3783091

[B45] Qin R. Wang J. Chao C. Yu J. Copeland L. Wang S.. (2021). RS5 produced more butyric acid through regulating the microbial community of human gut microbiota. J. Agric. Food Chem. 69, 3209–3218. 10.1021/acs.jafc.0c08187 33630575

[B46] Radjabzadeh D. Bosch J. A. Uitterlinden A. G. Zwinderman A. H. Ikram M. A. van Meurs J. B. J.. (2022). Gut microbiome-wide association study of depressive symptoms. Nat. Commun. 13, 7128. 10.1038/s41467-022-34502-3 36473852 PMC9726982

[B47] Robaire B. Henderson N. A. (2006). Actions of 5alpha-reductase inhibitors on the epididymis. Mol. Cell Endocrinol. 250, 190–195. 10.1016/j.mce.2005.12.044 16476520

[B48] Salas-Huetos A. Maghsoumi-Norouzabad L. James E. R. Carrell D. T. Aston K. I. Jenkins T. G.. (2021). Male adiposity, sperm parameters and reproductive hormones: an updated systematic review and collaborative meta-analysis. Obes. Rev. 22, e13082. 10.1111/obr.13082 32705766

[B49] Sharma R. Harlev A. Agarwal A. Esteves S. C. (2016). Cigarette smoking and semen quality: a new meta-analysis examining the effect of the 2010 world health organization laboratory methods for the examination of human semen. Eur. Urol. 70, 635–645. 10.1016/j.eururo.2016.04.010 27113031

[B50] Sharon G. Sampson T. R. Geschwind D. H. Mazmanian S. K. (2016). The central nervous system and the gut microbiome. Cell. 167, 915–932. 10.1016/j.cell.2016.10.027 27814521 PMC5127403

[B51] Sun Z. Y. Yu S. Tian Y. Han B. Q. Zhao Y. Li Y. Q.. (2022). Chestnut polysaccharides restore impaired spermatogenesis by adjusting gut microbiota and the intestinal structure. Food Funct. 13, 425–436. 10.1039/D1FO03145G 34913451

[B52] Thaiss C. A. Zmora N. Levy M. Elinav E. (2016). The microbiome and innate immunity. Nature. 535, 65–74. 10.1038/nature18847 27383981

[B53] Verbanck M. Chen C. Y. Neale B. Do R. (2018). Detection of widespread horizontal pleiotropy in causal relationships inferred from Mendelian randomization between complex traits and diseases. Nat. Genet. 50, 693–698. 10.1038/s41588-018-0099-7 29686387 PMC6083837

[B54] Verhoeven J. Keller D. Verbruggen S. Abboud K. Y. Venema K. A. (2021). blend of 3 mushrooms dose-dependently increases butyrate production by the gut microbiota. Benef. Microbes. 12, 601–612. 10.3920/BM2021.0015 34590532

[B55] Wang Y. Xie Z. (2022). Exploring the role of gut microbiome in male reproduction. Andrology. 10, 441–450. 10.1111/andr.13143 34918486

[B56] Wu G. D. Chen J. Hoffmann C. Bittinger K. Chen Y. Y. Keilbaugh S. A.. (2011). Linking long-term dietary patterns with gut microbial enterotypes. Science. 334, 105–108. 10.1126/science.1208344 21885731 PMC3368382

[B57] Xiang K. Wang P. Xu Z. Hu Y. Q. He Y. S. Chen Y.. (2021). Causal effects of gut microbiome on systemic lupus erythematosus: a two-sample mendelian randomization study. Front. Immunol. 12, 667097. 10.3389/fimmu.2021.667097 34557183 PMC8453215

[B58] Xue H. Shen X. Pan W. (2021). Constrained maximum likelihood-based Mendelian randomization robust to both correlated and uncorrelated pleiotropic effects. Am. J. Hum. Genet. 108, 1251–1269. 10.1016/j.ajhg.2021.05.014 34214446 PMC8322939

[B59] Yan X. Feng Y. Hao Y. Zhong R. Jiang Y. Tang X.. (2022). Gut-testis axis: microbiota prime metabolome to increase sperm quality in young type 2 diabetes. Microbiol. Spectr. 10, e0142322. 10.1128/spectrum.01423-22 36214691 PMC9603910

[B60] Yang M. Luo P. Zhang F. Xu K. Feng R. Xu P.. (2022). Large-scale correlation analysis of deep venous thrombosis and gut microbiota. Front. Cardiovasc. Med. 9, 1025918. 10.3389/fcvm.2022.1025918 36419497 PMC9677955

[B61] Yang M. Wan X. Zheng H. Xu K. Xie J. Yu H.. (2023). No evidence of a genetic causal relationship between ankylosing spondylitis and gut microbiota: a two-sample mendelian randomization study. Nutrients. 15, 57. 10.3390/nu15041057 36839415 PMC9965834

[B62] Zhang T. Sun P. Geng Q. Fan H. Gong Y. Hu Y.. (2022). Disrupted spermatogenesis in a metabolic syndrome model: the role of vitamin A metabolism in the gut-testis axis. Gut. 71, 78–87. 10.1136/gutjnl-2020-323347 33504491 PMC8666830

[B63] Zhao Q. Huang J. F. Cheng Y. Dai M. Y. Zhu W. F. Yang X. W.. (2021). Polyamine metabolism links gut microbiota and testicular dysfunction. Microbiome 9, 224. 10.1186/s40168-021-01157-z 34758869 PMC8582214

[B64] Zhao T. X. Wei Y. X. Wang J. K. Han L. D. Sun M. Wu Y. H.. (2020a). The gut-microbiota-testis axis mediated by the activation of the Nrf2 antioxidant pathway is related to prepuberal steroidogenesis disorders induced by di-(2-ethylhexyl) phthalate. Environ. Sci. Pollut. Res. Int. 27, 35261–35271. 10.1007/s11356-020-09854-2 32588312

[B65] Zhao Y. Zhang P. Ge W. Feng Y. Li L. Sun Z.. (2020b). Alginate oligosaccharides improve germ cell development and testicular microenvironment to rescue busulfan disrupted spermatogenesis. Theranostics. 10, 3308–3324. 10.7150/thno.43189 32194870 PMC7053202

