# Supplementary material for: Male infertility risk and gut microbiota: a Mendelian randomization study
Source: Front Microbiol. 2023 Sep 26;14:1228693. doi: 10.3389/fmicb.2023.1228693 (PMC10562550; doi:10.3389/fmicb.2023.1228693)
Supplement: Supplementary file 1 [file Data_Sheet_1.docx]

Supplementary Material

# Supplementary Figures and Tables

## Supplementary Figure

##
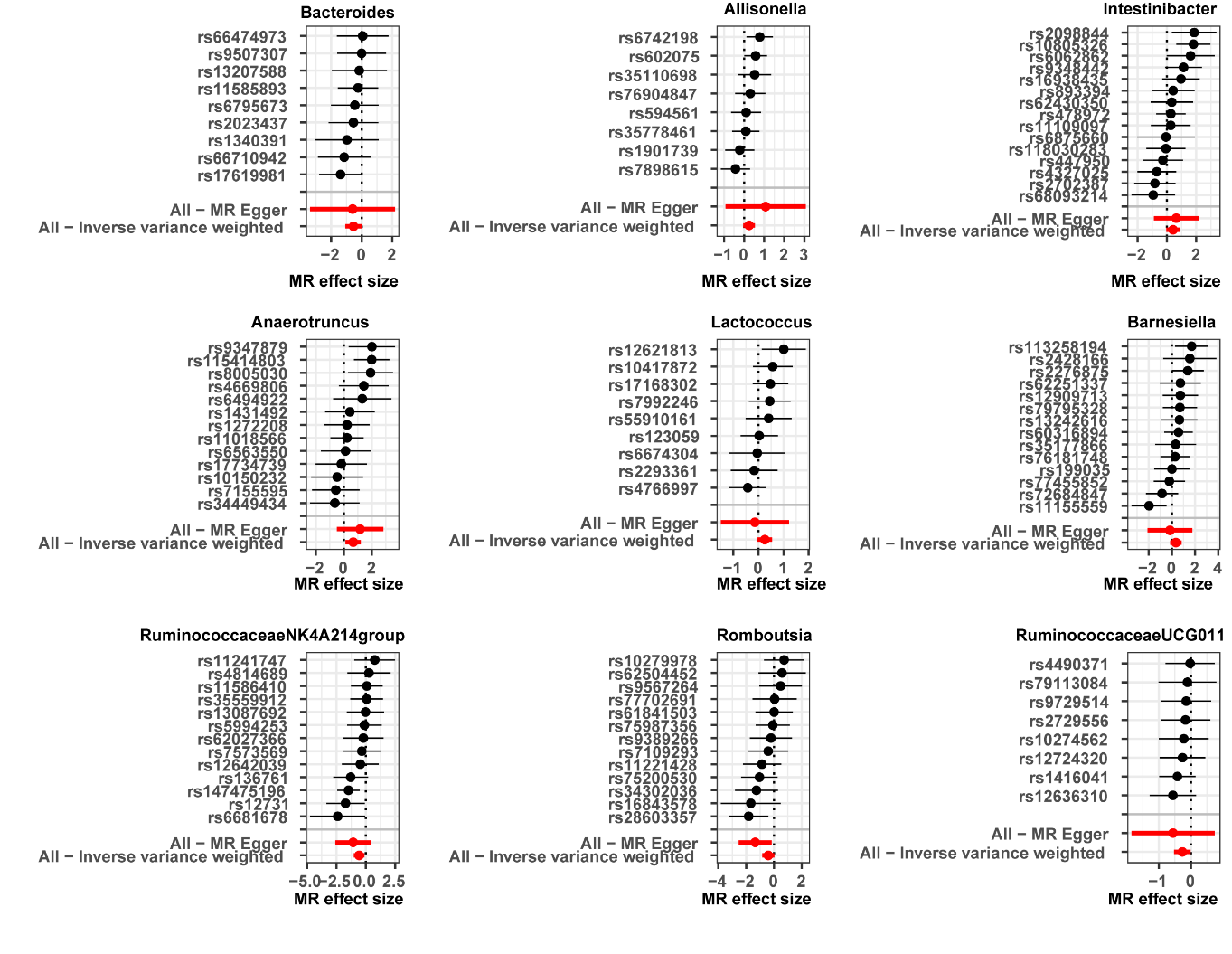


**Supplementary Figure 1.** Forest plots for the casual association between gut microbiota and male infertility


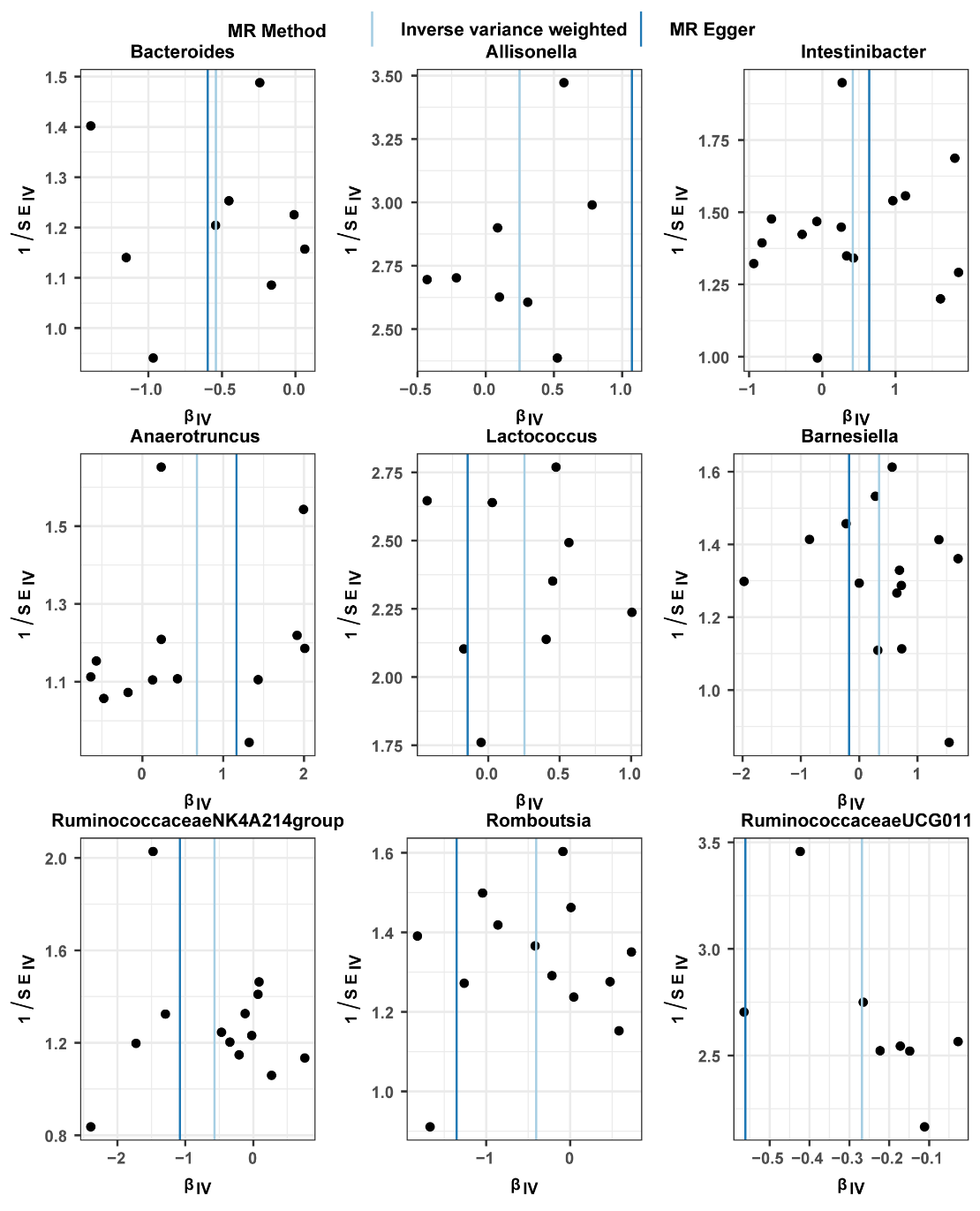
.

**Supplementary Figure 2.** Funnel plots for the casual association between gut microbiota and male infertility

## Supplementary table

| **Supplementary Table 1. All the IVs for MR analysis.** |  |  |  |  |  |  |  |
| --- | --- | --- | --- | --- | --- | --- | --- |
| **Bacterial taxa (exposure)** |  | **Nsnp** | **Methods** | **Beta** | **SE** | **P value** | **OR（95% CI）** |
| Actinomyces |  | 7 | IVW | 0.33 | 0.27 | 0.214 | 1.4(0.82-2.37) |
| Actinomyces |  | 7 | MR-Egger | 0.32 | 0.72 | 0.672 | 1.38(0.34-5.6) |
| Actinomyces |  | 7 | WM | 0.42 | 0.28 | 0.132 | 1.52(0.88-2.63) |
| Actinomyces |  | 7 | ML | 0.35 | 0.2 | 0.082 | 1.42(0.96-2.11) |
| Actinomyces |  | 7 | cML-MA-BIC | 0.3 | 0.22 | 0.172 | 1.36(0.88-2.1) |
| Adlercreutzia |  | 8 | IVW | 0.22 | 0.21 | 0.304 | 1.24(0.82-1.89) |
| Adlercreutzia |  | 8 | MR-Egger | -1.22 | 0.95 | 0.246 | 0.29(0.05-1.89) |
| Adlercreutzia |  | 8 | WM | 0.08 | 0.3 | 0.794 | 1.08(0.6-1.95) |
| Adlercreutzia |  | 8 | ML | 0.23 | 0.22 | 0.296 | 1.26(0.82-1.93) |
| Adlercreutzia |  | 8 | cML-MA-BIC | 0.25 | 0.23 | 0.271 | 1.29(0.82-2.03) |
| Akkermansia |  | 11 | IVW | 0.03 | 0.22 | 0.89 | 1.03(0.67-1.58) |
| Akkermansia |  | 11 | MR-Egger | 0.23 | 0.78 | 0.775 | 1.26(0.27-5.74) |
| Akkermansia |  | 11 | WM | 0.24 | 0.28 | 0.387 | 1.27(0.74-2.18) |
| Akkermansia |  | 11 | ML | 0.03 | 0.21 | 0.877 | 1.03(0.69-1.56) |
| Akkermansia |  | 11 | cML-MA-BIC | 0.05 | 0.22 | 0.808 | 1.05(0.69-1.61) |
| Alistipes |  | 13 | IVW | -0.37 | 0.26 | 0.148 | 0.69(0.42-1.14) |
| Alistipes |  | 13 | MR-Egger | -0.43 | 1.23 | 0.731 | 0.65(0.06-7.17) |
| Alistipes |  | 13 | WM | -0.2 | 0.35 | 0.579 | 0.82(0.41-1.64) |
| Alistipes |  | 13 | ML | -0.38 | 0.26 | 0.144 | 0.68(0.41-1.14) |
| Alistipes |  | 13 | cML-MA-BIC | -0.36 | 0.27 | 0.172 | 0.69(0.41-1.17) |
| Allisonella |  | 8 | IVW | 0.25 | 0.15 | 0.091 | 1.28(0.96-1.71) |
| Allisonella |  | 8 | MR-Egger | 1.07 | 1.02 | 0.333 | 2.92(0.4-21.46) |
| Allisonella |  | 8 | WM | 0.21 | 0.18 | 0.241 | 1.24(0.87-1.78) |
| Allisonella |  | 8 | ML | 0.26 | 0.13 | 0.045 | 1.3(1.01-1.68) |
| Allisonella |  | 8 | cML-MA-BIC | 0.24 | 0.14 | 0.089 | 1.27(0.96-1.67) |
| Alloprevotella |  | 6 | IVW | 0.2 | 0.17 | 0.231 | 1.22(0.88-1.7) |
| Alloprevotella |  | 6 | MR-Egger | 1.89 | 1.52 | 0.281 | 6.65(0.34-130.98) |
| Alloprevotella |  | 6 | WM | 0.18 | 0.19 | 0.339 | 1.2(0.82-1.76) |
| Alloprevotella |  | 6 | ML | 0.21 | 0.15 | 0.163 | 1.23(0.92-1.66) |
| Alloprevotella |  | 6 | cML-MA-BIC | 0.18 | 0.16 | 0.266 | 1.2(0.87-1.65) |
| Anaerofilum |  | 10 | IVW | -0.35 | 0.21 | 0.097 | 0.7(0.46-1.07) |
| Anaerofilum |  | 10 | MR-Egger | -0.8 | 1.22 | 0.531 | 0.45(0.04-4.93) |
| Anaerofilum |  | 10 | WM | -0.24 | 0.21 | 0.257 | 0.79(0.52-1.19) |
| Anaerofilum |  | 10 | ML | -0.38 | 0.15 | 0.012 | 0.68(0.5-0.92) |
| Anaerofilum |  | 10 | cML-MA-BIC | -0.2 | 0.18 | 0.285 | 0.82(0.57-1.18) |
| Anaerostipes |  | 13 | IVW | 0.21 | 0.24 | 0.379 | 1.23(0.77-1.97) |
| Anaerostipes |  | 13 | MR-Egger | 1 | 0.87 | 0.272 | 2.73(0.5-14.99) |
| Anaerostipes |  | 13 | WM | 0.25 | 0.33 | 0.451 | 1.28(0.67-2.46) |
| Anaerostipes |  | 13 | ML | 0.22 | 0.24 | 0.372 | 1.24(0.77-2) |
| Anaerostipes |  | 13 | cML-MA-BIC | 0.27 | 0.26 | 0.296 | 1.31(0.79-2.19) |
| Anaerotruncus |  | 13 | IVW | 0.67 | 0.28 | 0.016 | 1.96(1.13-3.4) |
| Anaerotruncus |  | 13 | MR-Egger | 1.16 | 0.85 | 0.196 | 3.21(0.61-16.86) |
| Anaerotruncus |  | 13 | WM | 0.23 | 0.36 | 0.513 | 1.26(0.63-2.54) |
| Anaerotruncus |  | 13 | ML | 0.7 | 0.24 | 0.004 | 2.01(1.26-3.2) |
| Anaerotruncus |  | 13 | cML-MA-BIC | 0.61 | 0.29 | 0.033 | 1.84(1.05-3.24) |
| Bacteroides |  | 9 | IVW | -0.54 | 0.27 | 0.048 | 0.58(0.34-0.99) |
| Bacteroides |  | 9 | MR-Egger | -0.6 | 1.42 | 0.686 | 0.55(0.03-8.88) |
| Bacteroides |  | 9 | WM | -0.39 | 0.35 | 0.263 | 0.68(0.34-1.34) |
| Bacteroides |  | 9 | ML | -0.54 | 0.28 | 0.052 | 0.58(0.34-1.01) |
| Bacteroides |  | 9 | cML-MA-BIC | -0.53 | 0.28 | 0.062 | 0.59(0.34-1.03) |
| Barnesiella |  | 14 | IVW | 0.34 | 0.25 | 0.175 | 1.41(0.86-2.31) |
| Barnesiella |  | 14 | MR-Egger | -0.17 | 0.99 | 0.863 | 0.84(0.12-5.8) |
| Barnesiella |  | 14 | WM | 0.53 | 0.3 | 0.072 | 1.7(0.95-3.03) |
| Barnesiella |  | 14 | ML | 0.35 | 0.21 | 0.092 | 1.42(0.94-2.15) |
| Barnesiella |  | 14 | cML-MA-BIC | 0.56 | 0.23 | 0.017 | 1.75(1.11-2.76) |
| Bifidobacterium |  | 13 | IVW | -0.2 | 0.18 | 0.273 | 0.82(0.57-1.17) |
| Bifidobacterium |  | 13 | MR-Egger | 0.09 | 0.47 | 0.858 | 1.09(0.44-2.72) |
| Bifidobacterium |  | 13 | WM | 0.08 | 0.26 | 0.75 | 1.09(0.65-1.81) |
| Bifidobacterium |  | 13 | ML | -0.21 | 0.19 | 0.272 | 0.81(0.57-1.17) |
| Bifidobacterium |  | 13 | cML-MA-BIC | -0.19 | 0.19 | 0.337 | 0.83(0.57-1.21) |
| Bilophila |  | 13 | IVW | 0.11 | 0.26 | 0.678 | 1.11(0.67-1.84) |
| Bilophila |  | 13 | MR-Egger | 0.22 | 1.33 | 0.869 | 1.25(0.09-17.1) |
| Bilophila |  | 13 | WM | 0.45 | 0.31 | 0.15 | 1.57(0.85-2.91) |
| Bilophila |  | 13 | ML | 0.11 | 0.22 | 0.608 | 1.12(0.73-1.73) |
| Bilophila |  | 13 | cML-MA-BIC | 0.26 | 0.27 | 0.343 | 1.29(0.76-2.19) |
| Blautia |  | 13 | IVW | 0.18 | 0.28 | 0.535 | 1.19(0.68-2.08) |
| Blautia |  | 13 | MR-Egger | 0.65 | 0.77 | 0.419 | 1.91(0.42-8.64) |
| Blautia |  | 13 | WM | 0.18 | 0.34 | 0.601 | 1.2(0.61-2.34) |
| Blautia |  | 13 | ML | 0.18 | 0.25 | 0.46 | 1.2(0.74-1.95) |
| Blautia |  | 13 | cML-MA-BIC | 0.01 | 0.28 | 0.965 | 1.01(0.59-1.75) |
| Butyricicoccus |  | 8 | IVW | -0.06 | 0.32 | 0.857 | 0.94(0.51-1.76) |
| Butyricicoccus |  | 8 | MR-Egger | 0.63 | 0.59 | 0.325 | 1.88(0.59-5.96) |
| Butyricicoccus |  | 8 | WM | 0.22 | 0.35 | 0.529 | 1.24(0.63-2.46) |
| Butyricicoccus |  | 8 | ML | -0.06 | 0.27 | 0.822 | 0.94(0.56-1.59) |
| Butyricicoccus |  | 8 | cML-MA-BIC | 0 | 0.29 | 0.99 | 1(0.57-1.76) |
| Butyricimonas |  | 13 | IVW | 0.17 | 0.21 | 0.422 | 1.18(0.79-1.78) |
| Butyricimonas |  | 13 | MR-Egger | -1.09 | 0.7 | 0.147 | 0.34(0.08-1.32) |
| Butyricimonas |  | 13 | WM | 0.15 | 0.28 | 0.603 | 1.16(0.67-2) |
| Butyricimonas |  | 13 | ML | 0.17 | 0.2 | 0.392 | 1.19(0.8-1.76) |
| Butyricimonas |  | 13 | cML-MA-BIC | 0.12 | 0.22 | 0.595 | 1.13(0.73-1.74) |
| Butyrivibrio |  | 15 | IVW | -0.07 | 0.12 | 0.564 | 0.93(0.74-1.18) |
| Butyrivibrio |  | 15 | MR-Egger | -0.17 | 0.52 | 0.754 | 0.85(0.3-2.36) |
| Butyrivibrio |  | 15 | WM | 0.01 | 0.13 | 0.95 | 1.01(0.78-1.31) |
| Butyrivibrio |  | 15 | ML | -0.07 | 0.1 | 0.496 | 0.94(0.77-1.13) |
| Butyrivibrio |  | 15 | cML-MA-BIC | -0.01 | 0.11 | 0.914 | 0.99(0.8-1.22) |
| CandidatusSoleaferrea |  | 10 | IVW | 0.12 | 0.16 | 0.476 | 1.12(0.81-1.55) |
| CandidatusSoleaferrea |  | 10 | MR-Egger | -0.21 | 1.87 | 0.914 | 0.81(0.02-31.47) |
| CandidatusSoleaferrea |  | 10 | WM | 0.23 | 0.22 | 0.28 | 1.26(0.83-1.93) |
| CandidatusSoleaferrea |  | 10 | ML | 0.12 | 0.16 | 0.458 | 1.13(0.82-1.56) |
| CandidatusSoleaferrea |  | 10 | cML-MA-BIC | 0.1 | 0.17 | 0.545 | 1.11(0.79-1.55) |
| Catenibacterium |  | 5 | IVW | 0.06 | 0.28 | 0.822 | 1.06(0.62-1.83) |
| Catenibacterium |  | 5 | MR-Egger | -1.62 | 2.81 | 0.605 | 0.2(0-48.62) |
| Catenibacterium |  | 5 | WM | -0.2 | 0.28 | 0.483 | 0.82(0.47-1.42) |
| Catenibacterium |  | 5 | ML | 0.07 | 0.18 | 0.706 | 1.07(0.75-1.53) |
| Catenibacterium |  | 5 | cML-MA-BIC | -0.07 | 0.26 | 0.789 | 0.93(0.56-1.55) |
| ChristensenellaceaeR.7group |  | 10 | IVW | -0.26 | 0.39 | 0.511 | 0.77(0.36-1.66) |
| ChristensenellaceaeR.7group |  | 10 | MR-Egger | 0.58 | 1.25 | 0.654 | 1.79(0.15-20.94) |
| ChristensenellaceaeR.7group |  | 10 | WM | -0.31 | 0.38 | 0.409 | 0.73(0.35-1.53) |
| ChristensenellaceaeR.7group |  | 10 | ML | -0.27 | 0.28 | 0.336 | 0.77(0.45-1.32) |
| ChristensenellaceaeR.7group |  | 10 | cML-MA-BIC | -0.25 | 0.3 | 0.406 | 0.78(0.43-1.4) |
| Clostridiuminnocuumgroup |  | 9 | IVW | 0.2 | 0.13 | 0.141 | 1.22(0.94-1.58) |
| Clostridiuminnocuumgroup |  | 9 | MR-Egger | 0.9 | 0.64 | 0.206 | 2.45(0.69-8.65) |
| Clostridiuminnocuumgroup |  | 9 | WM | 0.11 | 0.18 | 0.531 | 1.12(0.79-1.6) |
| Clostridiuminnocuumgroup |  | 9 | ML | 0.2 | 0.14 | 0.134 | 1.23(0.94-1.6) |
| Clostridiuminnocuumgroup |  | 9 | cML-MA-BIC | 0.15 | 0.15 | 0.303 | 1.17(0.87-1.56) |
| Clostridiumsensustricto1 |  | 7 | IVW | -0.12 | 0.24 | 0.624 | 0.89(0.55-1.43) |
| Clostridiumsensustricto1 |  | 7 | MR-Egger | -0.72 | 0.63 | 0.304 | 0.49(0.14-1.67) |
| Clostridiumsensustricto1 |  | 7 | WM | -0.29 | 0.3 | 0.343 | 0.75(0.42-1.36) |
| Clostridiumsensustricto1 |  | 7 | ML | -0.12 | 0.25 | 0.622 | 0.89(0.55-1.43) |
| Clostridiumsensustricto1 |  | 7 | cML-MA-BIC | -0.13 | 0.25 | 0.6 | 0.88(0.54-1.43) |
| Collinsella |  | 9 | IVW | 0.03 | 0.28 | 0.913 | 1.03(0.6-1.78) |
| Collinsella |  | 9 | MR-Egger | -1.05 | 1.04 | 0.346 | 0.35(0.05-2.68) |
| Collinsella |  | 9 | WM | -0.26 | 0.37 | 0.473 | 0.77(0.37-1.58) |
| Collinsella |  | 9 | ML | 0.03 | 0.28 | 0.912 | 1.03(0.59-1.79) |
| Collinsella |  | 9 | cML-MA-BIC | 0 | 0.29 | 0.998 | 1(0.56-1.77) |
| Coprobacter |  | 11 | IVW | 0.23 | 0.16 | 0.15 | 1.26(0.92-1.72) |
| Coprobacter |  | 11 | MR-Egger | 1.4 | 0.62 | 0.05 | 4.07(1.2-13.77) |
| Coprobacter |  | 11 | WM | 0.4 | 0.22 | 0.071 | 1.49(0.97-2.3) |
| Coprobacter |  | 11 | ML | 0.24 | 0.16 | 0.145 | 1.27(0.92-1.75) |
| Coprobacter |  | 11 | cML-MA-BIC | 0.27 | 0.17 | 0.12 | 1.31(0.93-1.84) |
| Coprococcus1 |  | 12 | IVW | -0.13 | 0.25 | 0.598 | 0.88(0.54-1.43) |
| Coprococcus1 |  | 12 | MR-Egger | -0.12 | 0.66 | 0.859 | 0.89(0.24-3.25) |
| Coprococcus1 |  | 12 | WM | -0.12 | 0.33 | 0.727 | 0.89(0.47-1.7) |
| Coprococcus1 |  | 12 | ML | -0.13 | 0.23 | 0.566 | 0.88(0.56-1.37) |
| Coprococcus1 |  | 12 | cML-MA-BIC | -0.05 | 0.26 | 0.835 | 0.95(0.57-1.58) |
| Coprococcus2 |  | 8 | IVW | -0.17 | 0.25 | 0.508 | 0.85(0.52-1.38) |
| Coprococcus2 |  | 8 | MR-Egger | -2.07 | 1.97 | 0.333 | 0.13(0-5.97) |
| Coprococcus2 |  | 8 | WM | -0.21 | 0.31 | 0.492 | 0.81(0.44-1.48) |
| Coprococcus2 |  | 8 | ML | -0.17 | 0.25 | 0.505 | 0.85(0.52-1.39) |
| Coprococcus2 |  | 8 | cML-MA-BIC | -0.17 | 0.25 | 0.496 | 0.84(0.51-1.38) |
| Coprococcus3 |  | 9 | IVW | 0.21 | 0.37 | 0.574 | 1.23(0.59-2.56) |
| Coprococcus3 |  | 9 | MR-Egger | 2.1 | 2.13 | 0.356 | 8.17(0.13-527.73) |
| Coprococcus3 |  | 9 | WM | 0.64 | 0.43 | 0.139 | 1.89(0.81-4.39) |
| Coprococcus3 |  | 9 | ML | 0.22 | 0.3 | 0.451 | 1.25(0.7-2.23) |
| Coprococcus3 |  | 9 | cML-MA-BIC | 0.19 | 0.31 | 0.541 | 1.21(0.65-2.25) |
| DefluviitaleaceaeUCG011 |  | 9 | IVW | 0.1 | 0.24 | 0.689 | 1.1(0.69-1.76) |
| DefluviitaleaceaeUCG011 |  | 9 | MR-Egger | 1 | 0.85 | 0.28 | 2.71(0.51-14.47) |
| DefluviitaleaceaeUCG011 |  | 9 | WM | -0.07 | 0.3 | 0.806 | 0.93(0.52-1.67) |
| DefluviitaleaceaeUCG011 |  | 9 | ML | 0.1 | 0.21 | 0.621 | 1.11(0.74-1.67) |
| DefluviitaleaceaeUCG011 |  | 9 | cML-MA-BIC | 0.05 | 0.23 | 0.823 | 1.05(0.67-1.64) |
| Desulfovibrio |  | 10 | IVW | 0.14 | 0.24 | 0.551 | 1.16(0.72-1.86) |
| Desulfovibrio |  | 10 | MR-Egger | -0.23 | 0.75 | 0.765 | 0.79(0.18-3.44) |
| Desulfovibrio |  | 10 | WM | 0.14 | 0.27 | 0.595 | 1.15(0.68-1.95) |
| Desulfovibrio |  | 10 | ML | 0.15 | 0.2 | 0.449 | 1.17(0.78-1.74) |
| Desulfovibrio |  | 10 | cML-MA-BIC | 0.24 | 0.23 | 0.303 | 1.27(0.81-1.99) |
| Dialister |  | 11 | IVW | 0.05 | 0.21 | 0.823 | 1.05(0.69-1.59) |
| Dialister |  | 11 | MR-Egger | 0.79 | 0.87 | 0.389 | 2.2(0.4-12.08) |
| Dialister |  | 11 | WM | 0.08 | 0.27 | 0.769 | 1.08(0.64-1.83) |
| Dialister |  | 11 | ML | 0.05 | 0.22 | 0.822 | 1.05(0.69-1.6) |
| Dialister |  | 11 | cML-MA-BIC | 0.06 | 0.22 | 0.781 | 1.06(0.69-1.63) |
| Dorea |  | 10 | IVW | 0.17 | 0.27 | 0.547 | 1.18(0.69-2.02) |
| Dorea |  | 10 | MR-Egger | -0.09 | 0.77 | 0.913 | 0.92(0.2-4.12) |
| Dorea |  | 10 | WM | 0.02 | 0.37 | 0.961 | 1.02(0.49-2.11) |
| Dorea |  | 10 | ML | 0.17 | 0.28 | 0.542 | 1.19(0.69-2.05) |
| Dorea |  | 10 | cML-MA-BIC | 0.14 | 0.29 | 0.635 | 1.15(0.65-2.02) |
| Eggerthella |  | 10 | IVW | -0.04 | 0.15 | 0.791 | 0.96(0.71-1.29) |
| Eggerthella |  | 10 | MR-Egger | -0.96 | 0.7 | 0.206 | 0.38(0.1-1.5) |
| Eggerthella |  | 10 | WM | -0.21 | 0.21 | 0.317 | 0.81(0.54-1.22) |
| Eggerthella |  | 10 | ML | -0.04 | 0.15 | 0.788 | 0.96(0.71-1.3) |
| Eggerthella |  | 10 | cML-MA-BIC | -0.06 | 0.16 | 0.7 | 0.94(0.68-1.29) |
| Eisenbergiella |  | 11 | IVW | -0.01 | 0.17 | 0.944 | 0.99(0.7-1.38) |
| Eisenbergiella |  | 11 | MR-Egger | -1.43 | 1.25 | 0.282 | 0.24(0.02-2.78) |
| Eisenbergiella |  | 11 | WM | -0.09 | 0.21 | 0.684 | 0.92(0.6-1.39) |
| Eisenbergiella |  | 11 | ML | -0.01 | 0.15 | 0.933 | 0.99(0.74-1.33) |
| Eisenbergiella |  | 11 | cML-MA-BIC | 0.01 | 0.16 | 0.944 | 1.01(0.74-1.38) |
| Enterorhabdus |  | 6 | IVW | -0.23 | 0.23 | 0.326 | 0.79(0.5-1.26) |
| Enterorhabdus |  | 6 | MR-Egger | 0.31 | 0.62 | 0.649 | 1.36(0.4-4.61) |
| Enterorhabdus |  | 6 | WM | -0.31 | 0.3 | 0.312 | 0.74(0.41-1.33) |
| Enterorhabdus |  | 6 | ML | -0.23 | 0.24 | 0.328 | 0.79(0.5-1.26) |
| Enterorhabdus |  | 6 | cML-MA-BIC | -0.22 | 0.24 | 0.357 | 0.8(0.5-1.29) |
| Erysipelatoclostridium |  | 15 | IVW | 0.18 | 0.16 | 0.276 | 1.19(0.87-1.64) |
| Erysipelatoclostridium |  | 15 | MR-Egger | 0.18 | 0.63 | 0.782 | 1.19(0.35-4.1) |
| Erysipelatoclostridium |  | 15 | WM | 0.17 | 0.22 | 0.438 | 1.18(0.77-1.82) |
| Erysipelatoclostridium |  | 15 | ML | 0.18 | 0.16 | 0.273 | 1.2(0.87-1.65) |
| Erysipelatoclostridium |  | 15 | cML-MA-BIC | 0.19 | 0.17 | 0.258 | 1.21(0.87-1.67) |
| ErysipelotrichaceaeUCG003 |  | 16 | IVW | -0.14 | 0.2 | 0.5 | 0.87(0.59-1.3) |
| ErysipelotrichaceaeUCG003 |  | 16 | MR-Egger | -0.47 | 0.56 | 0.417 | 0.62(0.21-1.88) |
| ErysipelotrichaceaeUCG003 |  | 16 | WM | -0.01 | 0.27 | 0.966 | 0.99(0.59-1.66) |
| ErysipelotrichaceaeUCG003 |  | 16 | ML | -0.13 | 0.19 | 0.474 | 0.87(0.6-1.26) |
| ErysipelotrichaceaeUCG003 |  | 16 | cML-MA-BIC | -0.1 | 0.2 | 0.606 | 0.9(0.61-1.33) |
| Escherichia.Shigella |  | 10 | IVW | -0.17 | 0.31 | 0.587 | 0.85(0.46-1.54) |
| Escherichia.Shigella |  | 10 | MR-Egger | -0.91 | 0.97 | 0.377 | 0.4(0.06-2.71) |
| Escherichia.Shigella |  | 10 | WM | 0.02 | 0.35 | 0.956 | 1.02(0.51-2.02) |
| Escherichia.Shigella |  | 10 | ML | -0.18 | 0.25 | 0.469 | 0.84(0.52-1.35) |
| Escherichia.Shigella |  | 10 | cML-MA-BIC | -0.09 | 0.28 | 0.759 | 0.92(0.53-1.6) |
| Eubacteriumbrachygroup |  | 10 | IVW | -0.11 | 0.13 | 0.424 | 0.9(0.69-1.17) |
| Eubacteriumbrachygroup |  | 10 | MR-Egger | -0.34 | 0.53 | 0.536 | 0.71(0.25-2) |
| Eubacteriumbrachygroup |  | 10 | WM | 0 | 0.17 | 0.984 | 1(0.71-1.4) |
| Eubacteriumbrachygroup |  | 10 | ML | -0.1 | 0.13 | 0.432 | 0.9(0.69-1.17) |
| Eubacteriumbrachygroup |  | 10 | cML-MA-BIC | -0.1 | 0.14 | 0.473 | 0.91(0.69-1.19) |
| Eubacteriumcoprostanoligenesgroup |  | 12 | IVW | 0 | 0.25 | 0.99 | 1(0.61-1.63) |
| Eubacteriumcoprostanoligenesgroup |  | 12 | MR-Egger | -0.5 | 0.97 | 0.618 | 0.61(0.09-4.04) |
| Eubacteriumcoprostanoligenesgroup |  | 12 | WM | -0.2 | 0.34 | 0.553 | 0.82(0.41-1.6) |
| Eubacteriumcoprostanoligenesgroup |  | 12 | ML | 0 | 0.26 | 0.99 | 1(0.6-1.64) |
| Eubacteriumcoprostanoligenesgroup |  | 12 | cML-MA-BIC | -0.03 | 0.26 | 0.91 | 0.97(0.58-1.63) |
| Eubacteriumeligensgroup |  | 7 | IVW | 0.35 | 0.39 | 0.377 | 1.41(0.66-3.04) |
| Eubacteriumeligensgroup |  | 7 | MR-Egger | -0.6 | 1.6 | 0.724 | 0.55(0.02-12.57) |
| Eubacteriumeligensgroup |  | 7 | WM | 0.26 | 0.44 | 0.565 | 1.29(0.54-3.09) |
| Eubacteriumeligensgroup |  | 7 | ML | 0.37 | 0.31 | 0.24 | 1.44(0.78-2.66) |
| Eubacteriumeligensgroup |  | 7 | cML-MA-BIC | 0.23 | 0.37 | 0.539 | 1.26(0.6-2.61) |
| Eubacteriumfissicatenagroup |  | 9 | IVW | -0.11 | 0.15 | 0.432 | 0.89(0.67-1.19) |
| Eubacteriumfissicatenagroup |  | 9 | MR-Egger | -1.37 | 0.73 | 0.105 | 0.25(0.06-1.07) |
| Eubacteriumfissicatenagroup |  | 9 | WM | 0.14 | 0.19 | 0.478 | 1.15(0.79-1.67) |
| Eubacteriumfissicatenagroup |  | 9 | ML | -0.11 | 0.14 | 0.425 | 0.89(0.67-1.18) |
| Eubacteriumfissicatenagroup |  | 9 | cML-MA-BIC | -0.07 | 0.16 | 0.644 | 0.93(0.68-1.27) |
| Eubacteriumhalliigroup |  | 16 | IVW | -0.2 | 0.18 | 0.265 | 0.82(0.58-1.16) |
| Eubacteriumhalliigroup |  | 16 | MR-Egger | -0.49 | 0.37 | 0.21 | 0.61(0.3-1.27) |
| Eubacteriumhalliigroup |  | 16 | WM | -0.1 | 0.24 | 0.677 | 0.9(0.56-1.45) |
| Eubacteriumhalliigroup |  | 16 | ML | -0.2 | 0.18 | 0.278 | 0.82(0.58-1.17) |
| Eubacteriumhalliigroup |  | 16 | cML-MA-BIC | -0.19 | 0.19 | 0.309 | 0.83(0.57-1.19) |
| Eubacteriumnodatumgroup |  | 11 | IVW | 0.08 | 0.12 | 0.489 | 1.08(0.86-1.36) |
| Eubacteriumnodatumgroup |  | 11 | MR-Egger | -0.1 | 0.51 | 0.844 | 0.9(0.33-2.47) |
| Eubacteriumnodatumgroup |  | 11 | WM | 0.19 | 0.15 | 0.214 | 1.2(0.9-1.61) |
| Eubacteriumnodatumgroup |  | 11 | ML | 0.08 | 0.12 | 0.48 | 1.09(0.86-1.37) |
| Eubacteriumnodatumgroup |  | 11 | cML-MA-BIC | 0.07 | 0.12 | 0.543 | 1.08(0.85-1.36) |
| Eubacteriumoxidoreducensgroup |  | 5 | IVW | 0.34 | 0.21 | 0.109 | 1.4(0.93-2.11) |
| Eubacteriumoxidoreducensgroup |  | 5 | MR-Egger | 0.23 | 0.79 | 0.786 | 1.26(0.27-5.89) |
| Eubacteriumoxidoreducensgroup |  | 5 | WM | 0.42 | 0.26 | 0.106 | 1.53(0.91-2.56) |
| Eubacteriumoxidoreducensgroup |  | 5 | ML | 0.34 | 0.21 | 0.112 | 1.4(0.92-2.13) |
| Eubacteriumoxidoreducensgroup |  | 5 | cML-MA-BIC | 0.34 | 0.22 | 0.111 | 1.41(0.92-2.15) |
| Eubacteriumrectalegroup |  | 8 | IVW | -0.58 | 0.33 | 0.078 | 0.56(0.3-1.07) |
| Eubacteriumrectalegroup |  | 8 | MR-Egger | -0.95 | 1.29 | 0.49 | 0.39(0.03-4.83) |
| Eubacteriumrectalegroup |  | 8 | WM | -0.5 | 0.41 | 0.222 | 0.61(0.27-1.35) |
| Eubacteriumrectalegroup |  | 8 | ML | -0.59 | 0.3 | 0.051 | 0.56(0.31-1) |
| Eubacteriumrectalegroup |  | 8 | cML-MA-BIC | -0.54 | 0.33 | 0.095 | 0.58(0.31-1.1) |
| Eubacteriumruminantiumgroup |  | 18 | IVW | 0.1 | 0.13 | 0.424 | 1.11(0.86-1.42) |
| Eubacteriumruminantiumgroup |  | 18 | MR-Egger | 0.25 | 0.42 | 0.559 | 1.29(0.56-2.95) |
| Eubacteriumruminantiumgroup |  | 18 | WM | 0.02 | 0.18 | 0.893 | 1.02(0.73-1.45) |
| Eubacteriumruminantiumgroup |  | 18 | ML | 0.11 | 0.13 | 0.405 | 1.11(0.87-1.43) |
| Eubacteriumruminantiumgroup |  | 18 | cML-MA-BIC | 0.09 | 0.13 | 0.472 | 1.1(0.85-1.42) |
| Eubacteriumventriosumgroup |  | 15 | IVW | -0.1 | 0.21 | 0.653 | 0.91(0.6-1.38) |
| Eubacteriumventriosumgroup |  | 15 | MR-Egger | 0.78 | 0.95 | 0.426 | 2.18(0.34-14.05) |
| Eubacteriumventriosumgroup |  | 15 | WM | -0.02 | 0.28 | 0.933 | 0.98(0.56-1.7) |
| Eubacteriumventriosumgroup |  | 15 | ML | -0.09 | 0.21 | 0.659 | 0.91(0.61-1.37) |
| Eubacteriumventriosumgroup |  | 15 | cML-MA-BIC | -0.05 | 0.22 | 0.817 | 0.95(0.62-1.46) |
| Eubacteriumxylanophilumgroup |  | 9 | IVW | 0.18 | 0.27 | 0.519 | 1.19(0.7-2.04) |
| Eubacteriumxylanophilumgroup |  | 9 | MR-Egger | 1.1 | 0.8 | 0.212 | 3.01(0.62-14.48) |
| Eubacteriumxylanophilumgroup |  | 9 | WM | 0.18 | 0.34 | 0.596 | 1.2(0.61-2.35) |
| Eubacteriumxylanophilumgroup |  | 9 | ML | 0.19 | 0.24 | 0.43 | 1.2(0.76-1.91) |
| Eubacteriumxylanophilumgroup |  | 9 | cML-MA-BIC | -0.03 | 0.29 | 0.918 | 0.97(0.55-1.72) |
| Faecalibacterium |  | 10 | IVW | 0.23 | 0.21 | 0.281 | 1.25(0.83-1.89) |
| Faecalibacterium |  | 10 | MR-Egger | -0.08 | 0.41 | 0.847 | 0.92(0.41-2.06) |
| Faecalibacterium |  | 10 | WM | 0.23 | 0.29 | 0.424 | 1.26(0.71-2.23) |
| Faecalibacterium |  | 10 | ML | 0.23 | 0.21 | 0.275 | 1.26(0.83-1.92) |
| Faecalibacterium |  | 10 | cML-MA-BIC | 0.21 | 0.22 | 0.341 | 1.23(0.8-1.89) |
| FamilyXIIIAD3011group |  | 13 | IVW | -0.14 | 0.21 | 0.519 | 0.87(0.57-1.32) |
| FamilyXIIIAD3011group |  | 13 | MR-Egger | -0.21 | 1.01 | 0.837 | 0.81(0.11-5.85) |
| FamilyXIIIAD3011group |  | 13 | WM | -0.14 | 0.28 | 0.621 | 0.87(0.51-1.5) |
| FamilyXIIIAD3011group |  | 13 | ML | -0.13 | 0.22 | 0.534 | 0.87(0.57-1.33) |
| FamilyXIIIAD3011group |  | 13 | cML-MA-BIC | -0.15 | 0.22 | 0.484 | 0.86(0.56-1.32) |
| FamilyXIIIUCG001 |  | 8 | IVW | -0.07 | 0.35 | 0.836 | 0.93(0.46-1.86) |
| FamilyXIIIUCG001 |  | 8 | MR-Egger | -0.82 | 1.14 | 0.502 | 0.44(0.05-4.16) |
| FamilyXIIIUCG001 |  | 8 | WM | -0.29 | 0.35 | 0.405 | 0.75(0.38-1.48) |
| FamilyXIIIUCG001 |  | 8 | ML | -0.08 | 0.26 | 0.765 | 0.93(0.56-1.54) |
| FamilyXIIIUCG001 |  | 8 | cML-MA-BIC | -0.33 | 0.32 | 0.302 | 0.72(0.38-1.35) |
| Flavonifractor |  | 5 | IVW | -0.11 | 0.29 | 0.705 | 0.9(0.51-1.58) |
| Flavonifractor |  | 5 | MR-Egger | -1.25 | 1.16 | 0.361 | 0.29(0.03-2.79) |
| Flavonifractor |  | 5 | WM | -0.35 | 0.38 | 0.351 | 0.7(0.33-1.48) |
| Flavonifractor |  | 5 | ML | -0.11 | 0.29 | 0.704 | 0.89(0.5-1.59) |
| Flavonifractor |  | 5 | cML-MA-BIC | -0.14 | 0.31 | 0.646 | 0.87(0.48-1.59) |
| Fusicatenibacter |  | 18 | IVW | -0.32 | 0.23 | 0.155 | 0.73(0.47-1.13) |
| Fusicatenibacter |  | 18 | MR-Egger | 0.77 | 0.83 | 0.366 | 2.16(0.43-10.88) |
| Fusicatenibacter |  | 18 | WM | -0.3 | 0.29 | 0.315 | 0.74(0.42-1.32) |
| Fusicatenibacter |  | 18 | ML | -0.32 | 0.21 | 0.119 | 0.72(0.48-1.09) |
| Fusicatenibacter |  | 18 | cML-MA-BIC | -0.24 | 0.23 | 0.293 | 0.79(0.5-1.23) |
| Gordonibacter |  | 12 | IVW | 0.12 | 0.12 | 0.311 | 1.12(0.9-1.41) |
| Gordonibacter |  | 12 | MR-Egger | -0.08 | 0.5 | 0.874 | 0.92(0.35-2.44) |
| Gordonibacter |  | 12 | WM | 0.19 | 0.16 | 0.232 | 1.2(0.89-1.63) |
| Gordonibacter |  | 12 | ML | 0.12 | 0.12 | 0.295 | 1.13(0.9-1.42) |
| Gordonibacter |  | 12 | cML-MA-BIC | 0.11 | 0.12 | 0.379 | 1.11(0.88-1.41) |
| Haemophilus |  | 9 | IVW | -0.13 | 0.18 | 0.475 | 0.88(0.62-1.25) |
| Haemophilus |  | 9 | MR-Egger | -0.36 | 0.4 | 0.398 | 0.7(0.32-1.52) |
| Haemophilus |  | 9 | WM | -0.14 | 0.23 | 0.545 | 0.87(0.55-1.37) |
| Haemophilus |  | 9 | ML | -0.13 | 0.18 | 0.471 | 0.88(0.62-1.25) |
| Haemophilus |  | 9 | cML-MA-BIC | -0.12 | 0.18 | 0.52 | 0.89(0.62-1.27) |
| Holdemanella |  | 11 | IVW | -0.13 | 0.23 | 0.562 | 0.88(0.56-1.37) |
| Holdemanella |  | 11 | MR-Egger | 0.28 | 0.68 | 0.684 | 1.33(0.35-5) |
| Holdemanella |  | 11 | WM | -0.13 | 0.24 | 0.604 | 0.88(0.55-1.42) |
| Holdemanella |  | 11 | ML | -0.13 | 0.16 | 0.404 | 0.88(0.64-1.2) |
| Holdemanella |  | 11 | cML-MA-BIC | -0.01 | 0.2 | 0.97 | 0.99(0.67-1.46) |
| Holdemania |  | 14 | IVW | 0.23 | 0.16 | 0.154 | 1.26(0.92-1.74) |
| Holdemania |  | 14 | MR-Egger | 0.74 | 0.49 | 0.154 | 2.1(0.81-5.43) |
| Holdemania |  | 14 | WM | 0.36 | 0.23 | 0.119 | 1.43(0.91-2.23) |
| Holdemania |  | 14 | ML | 0.24 | 0.17 | 0.15 | 1.27(0.92-1.76) |
| Holdemania |  | 14 | cML-MA-BIC | 0.22 | 0.18 | 0.217 | 1.24(0.88-1.75) |
| Howardella |  | 9 | IVW | 0 | 0.15 | 0.986 | 1(0.75-1.35) |
| Howardella |  | 9 | MR-Egger | 0.45 | 0.62 | 0.491 | 1.56(0.47-5.22) |
| Howardella |  | 9 | WM | 0.17 | 0.17 | 0.32 | 1.19(0.85-1.66) |
| Howardella |  | 9 | ML | 0 | 0.13 | 0.983 | 1(0.78-1.29) |
| Howardella |  | 9 | cML-MA-BIC | 0.03 | 0.13 | 0.84 | 1.03(0.79-1.34) |
| Hungatella |  | 5 | IVW | 0.08 | 0.19 | 0.684 | 1.08(0.75-1.56) |
| Hungatella |  | 5 | MR-Egger | 1.8 | 1.15 | 0.215 | 6.04(0.64-56.97) |
| Hungatella |  | 5 | WM | 0.15 | 0.24 | 0.537 | 1.16(0.72-1.88) |
| Hungatella |  | 5 | ML | 0.08 | 0.19 | 0.68 | 1.08(0.74-1.58) |
| Hungatella |  | 5 | cML-MA-BIC | 0.07 | 0.2 | 0.718 | 1.07(0.73-1.57) |
| Intestinibacter |  | 15 | IVW | 0.42 | 0.23 | 0.074 | 1.51(0.96-2.39) |
| Intestinibacter |  | 15 | MR-Egger | 0.64 | 0.78 | 0.429 | 1.89(0.41-8.79) |
| Intestinibacter |  | 15 | WM | 0.27 | 0.27 | 0.32 | 1.31(0.77-2.23) |
| Intestinibacter |  | 15 | ML | 0.43 | 0.19 | 0.021 | 1.53(1.07-2.2) |
| Intestinibacter |  | 15 | cML-MA-BIC | 0.32 | 0.22 | 0.154 | 1.37(0.89-2.12) |
| Intestinimonas |  | 16 | IVW | 0.03 | 0.17 | 0.88 | 1.03(0.73-1.44) |
| Intestinimonas |  | 16 | MR-Egger | -0.28 | 0.48 | 0.572 | 0.76(0.3-1.94) |
| Intestinimonas |  | 16 | WM | 0.07 | 0.24 | 0.763 | 1.07(0.67-1.72) |
| Intestinimonas |  | 16 | ML | 0.03 | 0.18 | 0.879 | 1.03(0.73-1.45) |
| Intestinimonas |  | 16 | cML-MA-BIC | 0.06 | 0.18 | 0.737 | 1.06(0.74-1.52) |
| Lachnoclostridium |  | 13 | IVW | 0.29 | 0.23 | 0.208 | 1.34(0.85-2.11) |
| Lachnoclostridium |  | 13 | MR-Egger | 0.15 | 0.79 | 0.853 | 1.16(0.25-5.44) |
| Lachnoclostridium |  | 13 | WM | 0.37 | 0.35 | 0.287 | 1.45(0.73-2.86) |
| Lachnoclostridium |  | 13 | ML | 0.3 | 0.24 | 0.205 | 1.35(0.85-2.15) |
| Lachnoclostridium |  | 13 | cML-MA-BIC | 0.36 | 0.25 | 0.159 | 1.43(0.87-2.35) |
| Lachnospira |  | 6 | IVW | 0.02 | 0.36 | 0.958 | 1.02(0.5-2.06) |
| Lachnospira |  | 6 | MR-Egger | 0.74 | 2.17 | 0.751 | 2.09(0.03-145.91) |
| Lachnospira |  | 6 | WM | -0.17 | 0.46 | 0.718 | 0.85(0.34-2.09) |
| Lachnospira |  | 6 | ML | 0.02 | 0.36 | 0.957 | 1.02(0.5-2.08) |
| Lachnospira |  | 6 | cML-MA-BIC | -0.03 | 0.38 | 0.931 | 0.97(0.46-2.05) |
| LachnospiraceaeFCS020group |  | 12 | IVW | -0.12 | 0.21 | 0.555 | 0.89(0.59-1.33) |
| LachnospiraceaeFCS020group |  | 12 | MR-Egger | -0.96 | 0.55 | 0.112 | 0.38(0.13-1.13) |
| LachnospiraceaeFCS020group |  | 12 | WM | -0.06 | 0.28 | 0.84 | 0.94(0.54-1.65) |
| LachnospiraceaeFCS020group |  | 12 | ML | -0.13 | 0.21 | 0.548 | 0.88(0.58-1.33) |
| LachnospiraceaeFCS020group |  | 12 | cML-MA-BIC | -0.06 | 0.23 | 0.798 | 0.94(0.6-1.49) |
| LachnospiraceaeNC2004group |  | 9 | IVW | 0.02 | 0.17 | 0.915 | 1.02(0.73-1.42) |
| LachnospiraceaeNC2004group |  | 9 | MR-Egger | -0.01 | 0.69 | 0.985 | 0.99(0.26-3.82) |
| LachnospiraceaeNC2004group |  | 9 | WM | -0.07 | 0.22 | 0.764 | 0.94(0.61-1.43) |
| LachnospiraceaeNC2004group |  | 9 | ML | 0.02 | 0.17 | 0.914 | 1.02(0.73-1.43) |
| LachnospiraceaeNC2004group |  | 9 | cML-MA-BIC | 0 | 0.18 | 0.994 | 1(0.71-1.42) |
| LachnospiraceaeND3007group |  | 3 | IVW | 0.2 | 0.49 | 0.687 | 1.22(0.47-3.18) |
| LachnospiraceaeND3007group |  | 3 | MR-Egger | -11.38 | 8.17 | 0.397 | 0(0-103.81) |
| LachnospiraceaeND3007group |  | 3 | WM | 0.65 | 0.65 | 0.319 | 1.91(0.53-6.82) |
| LachnospiraceaeND3007group |  | 3 | ML | 0.2 | 0.49 | 0.679 | 1.23(0.47-3.22) |
| LachnospiraceaeND3007group |  | 3 | cML-MA-BIC | 0.26 | 0.52 | 0.623 | 1.29(0.47-3.57) |
| LachnospiraceaeNK4A136group |  | 15 | IVW | -0.02 | 0.27 | 0.948 | 0.98(0.58-1.67) |
| LachnospiraceaeNK4A136group |  | 15 | MR-Egger | 0.72 | 0.51 | 0.184 | 2.05(0.75-5.62) |
| LachnospiraceaeNK4A136group |  | 15 | WM | 0.55 | 0.3 | 0.064 | 1.73(0.97-3.1) |
| LachnospiraceaeNK4A136group |  | 15 | ML | -0.02 | 0.2 | 0.92 | 0.98(0.66-1.45) |
| LachnospiraceaeNK4A136group |  | 15 | cML-MA-BIC | -0.3 | 0.35 | 0.388 | 0.74(0.37-1.47) |
| LachnospiraceaeUCG001 |  | 13 | IVW | -0.16 | 0.26 | 0.546 | 0.86(0.52-1.42) |
| LachnospiraceaeUCG001 |  | 13 | MR-Egger | 0.57 | 1.14 | 0.626 | 1.77(0.19-16.69) |
| LachnospiraceaeUCG001 |  | 13 | WM | -0.14 | 0.26 | 0.575 | 0.87(0.52-1.43) |
| LachnospiraceaeUCG001 |  | 13 | ML | -0.17 | 0.18 | 0.34 | 0.84(0.59-1.2) |
| LachnospiraceaeUCG001 |  | 13 | cML-MA-BIC | 0.11 | 0.22 | 0.604 | 1.12(0.73-1.72) |
| LachnospiraceaeUCG004 |  | 12 | IVW | -0.27 | 0.23 | 0.244 | 0.76(0.49-1.2) |
| LachnospiraceaeUCG004 |  | 12 | MR-Egger | -0.42 | 0.95 | 0.673 | 0.66(0.1-4.29) |
| LachnospiraceaeUCG004 |  | 12 | WM | -0.27 | 0.29 | 0.347 | 0.76(0.43-1.35) |
| LachnospiraceaeUCG004 |  | 12 | ML | -0.27 | 0.23 | 0.24 | 0.76(0.48-1.2) |
| LachnospiraceaeUCG004 |  | 12 | cML-MA-BIC | -0.27 | 0.24 | 0.258 | 0.77(0.48-1.22) |
| LachnospiraceaeUCG008 |  | 10 | IVW | 0 | 0.18 | 0.991 | 1(0.7-1.43) |
| LachnospiraceaeUCG008 |  | 10 | MR-Egger | 1.81 | 0.82 | 0.057 | 6.12(1.24-30.34) |
| LachnospiraceaeUCG008 |  | 10 | WM | -0.07 | 0.22 | 0.763 | 0.93(0.6-1.45) |
| LachnospiraceaeUCG008 |  | 10 | ML | 0 | 0.17 | 0.989 | 1(0.72-1.39) |
| LachnospiraceaeUCG008 |  | 10 | cML-MA-BIC | 0.11 | 0.18 | 0.547 | 1.12(0.78-1.59) |
| LachnospiraceaeUCG010 |  | 10 | IVW | -0.35 | 0.28 | 0.222 | 0.71(0.41-1.23) |
| LachnospiraceaeUCG010 |  | 10 | MR-Egger | -1.71 | 0.78 | 0.06 | 0.18(0.04-0.84) |
| LachnospiraceaeUCG010 |  | 10 | WM | -0.59 | 0.34 | 0.083 | 0.55(0.28-1.08) |
| LachnospiraceaeUCG010 |  | 10 | ML | -0.36 | 0.25 | 0.147 | 0.7(0.43-1.14) |
| LachnospiraceaeUCG010 |  | 10 | cML-MA-BIC | -0.41 | 0.27 | 0.127 | 0.66(0.39-1.12) |
| Lactobacillus |  | 8 | IVW | 0.17 | 0.18 | 0.345 | 1.18(0.84-1.67) |
| Lactobacillus |  | 8 | MR-Egger | -0.2 | 0.46 | 0.673 | 0.82(0.33-2.01) |
| Lactobacillus |  | 8 | WM | -0.07 | 0.23 | 0.754 | 0.93(0.6-1.45) |
| Lactobacillus |  | 8 | ML | 0.17 | 0.17 | 0.304 | 1.19(0.85-1.66) |
| Lactobacillus |  | 8 | cML-MA-BIC | 0.15 | 0.18 | 0.406 | 1.16(0.82-1.65) |
| Lactococcus |  | 9 | IVW | 0.25 | 0.15 | 0.085 | 1.29(0.97-1.72) |
| Lactococcus |  | 9 | MR-Egger | -0.14 | 0.69 | 0.841 | 0.87(0.22-3.36) |
| Lactococcus |  | 9 | WM | 0.41 | 0.2 | 0.039 | 1.51(1.02-2.22) |
| Lactococcus |  | 9 | ML | 0.26 | 0.15 | 0.071 | 1.3(0.98-1.73) |
| Lactococcus |  | 9 | cML-MA-BIC | 0.25 | 0.15 | 0.098 | 1.28(0.95-1.73) |
| Marvinbryantia |  | 10 | IVW | -0.13 | 0.23 | 0.567 | 0.88(0.56-1.38) |
| Marvinbryantia |  | 10 | MR-Egger | -0.95 | 0.91 | 0.329 | 0.39(0.06-2.32) |
| Marvinbryantia |  | 10 | WM | -0.19 | 0.32 | 0.56 | 0.83(0.45-1.55) |
| Marvinbryantia |  | 10 | ML | -0.14 | 0.23 | 0.559 | 0.87(0.55-1.38) |
| Marvinbryantia |  | 10 | cML-MA-BIC | -0.1 | 0.25 | 0.698 | 0.91(0.56-1.48) |
| Methanobrevibacter |  | 6 | IVW | -0.18 | 0.22 | 0.407 | 0.84(0.55-1.28) |
| Methanobrevibacter |  | 6 | MR-Egger | 0.07 | 0.9 | 0.945 | 1.07(0.18-6.24) |
| Methanobrevibacter |  | 6 | WM | -0.13 | 0.25 | 0.589 | 0.87(0.54-1.42) |
| Methanobrevibacter |  | 6 | ML | -0.19 | 0.18 | 0.283 | 0.82(0.58-1.17) |
| Methanobrevibacter |  | 6 | cML-MA-BIC | -0.16 | 0.2 | 0.43 | 0.85(0.58-1.26) |
| Odoribacter |  | 7 | IVW | -0.15 | 0.28 | 0.595 | 0.86(0.49-1.5) |
| Odoribacter |  | 7 | MR-Egger | -0.55 | 0.91 | 0.574 | 0.58(0.1-3.45) |
| Odoribacter |  | 7 | WM | -0.27 | 0.35 | 0.443 | 0.76(0.38-1.52) |
| Odoribacter |  | 7 | ML | -0.16 | 0.29 | 0.589 | 0.86(0.49-1.51) |
| Odoribacter |  | 7 | cML-MA-BIC | -0.12 | 0.3 | 0.683 | 0.88(0.49-1.59) |
| Olsenella |  | 11 | IVW | -0.03 | 0.13 | 0.831 | 0.97(0.75-1.26) |
| Olsenella |  | 11 | MR-Egger | 0.3 | 0.45 | 0.52 | 1.35(0.56-3.28) |
| Olsenella |  | 11 | WM | -0.1 | 0.17 | 0.574 | 0.91(0.65-1.27) |
| Olsenella |  | 11 | ML | -0.03 | 0.12 | 0.814 | 0.97(0.77-1.23) |
| Olsenella |  | 11 | cML-MA-BIC | -0.01 | 0.13 | 0.939 | 0.99(0.77-1.27) |
| Oscillibacter |  | 14 | IVW | 0.21 | 0.17 | 0.211 | 1.24(0.89-1.73) |
| Oscillibacter |  | 14 | MR-Egger | 0.07 | 0.67 | 0.92 | 1.07(0.29-4) |
| Oscillibacter |  | 14 | WM | 0.08 | 0.22 | 0.711 | 1.08(0.71-1.66) |
| Oscillibacter |  | 14 | ML | 0.22 | 0.16 | 0.172 | 1.25(0.91-1.71) |
| Oscillibacter |  | 14 | cML-MA-BIC | 0.13 | 0.18 | 0.465 | 1.14(0.8-1.64) |
| Oscillospira |  | 8 | IVW | -0.23 | 0.29 | 0.431 | 0.8(0.46-1.4) |
| Oscillospira |  | 8 | MR-Egger | 1.41 | 1.11 | 0.251 | 4.1(0.46-36.22) |
| Oscillospira |  | 8 | WM | -0.39 | 0.31 | 0.203 | 0.68(0.37-1.24) |
| Oscillospira |  | 8 | ML | -0.24 | 0.23 | 0.302 | 0.79(0.5-1.24) |
| Oscillospira |  | 8 | cML-MA-BIC | -0.42 | 0.26 | 0.109 | 0.66(0.39-1.1) |
| Oxalobacter |  | 11 | IVW | 0.17 | 0.13 | 0.185 | 1.18(0.92-1.51) |
| Oxalobacter |  | 11 | MR-Egger | -0.52 | 0.59 | 0.403 | 0.59(0.19-1.9) |
| Oxalobacter |  | 11 | WM | 0.21 | 0.18 | 0.238 | 1.24(0.87-1.76) |
| Oxalobacter |  | 11 | ML | 0.17 | 0.13 | 0.175 | 1.19(0.93-1.53) |
| Oxalobacter |  | 11 | cML-MA-BIC | 0.16 | 0.13 | 0.219 | 1.17(0.91-1.52) |
| Parabacteroides |  | 6 | IVW | -0.27 | 0.36 | 0.464 | 0.77(0.37-1.57) |
| Parabacteroides |  | 6 | MR-Egger | -0.58 | 1.24 | 0.664 | 0.56(0.05-6.32) |
| Parabacteroides |  | 6 | WM | -0.29 | 0.39 | 0.452 | 0.75(0.35-1.6) |
| Parabacteroides |  | 6 | ML | -0.28 | 0.29 | 0.341 | 0.76(0.43-1.34) |
| Parabacteroides |  | 6 | cML-MA-BIC | -0.23 | 0.31 | 0.451 | 0.79(0.43-1.45) |
| Paraprevotella |  | 13 | IVW | 0.06 | 0.14 | 0.653 | 1.06(0.81-1.4) |
| Paraprevotella |  | 13 | MR-Egger | 0.02 | 0.55 | 0.974 | 1.02(0.35-2.99) |
| Paraprevotella |  | 13 | WM | -0.15 | 0.19 | 0.421 | 0.86(0.6-1.24) |
| Paraprevotella |  | 13 | ML | 0.07 | 0.14 | 0.642 | 1.07(0.81-1.41) |
| Paraprevotella |  | 13 | cML-MA-BIC | 0.05 | 0.15 | 0.737 | 1.05(0.79-1.41) |
| Parasutterella |  | 14 | IVW | 0.17 | 0.2 | 0.383 | 1.19(0.81-1.75) |
| Parasutterella |  | 14 | MR-Egger | -0.28 | 0.55 | 0.623 | 0.76(0.26-2.23) |
| Parasutterella |  | 14 | WM | 0.27 | 0.25 | 0.282 | 1.31(0.8-2.13) |
| Parasutterella |  | 14 | ML | 0.18 | 0.18 | 0.306 | 1.2(0.85-1.69) |
| Parasutterella |  | 14 | cML-MA-BIC | 0.14 | 0.2 | 0.479 | 1.15(0.78-1.71) |
| Peptococcus |  | 12 | IVW | 0.17 | 0.13 | 0.209 | 1.18(0.91-1.54) |
| Peptococcus |  | 12 | MR-Egger | -0.52 | 0.51 | 0.333 | 0.59(0.22-1.62) |
| Peptococcus |  | 12 | WM | 0.12 | 0.18 | 0.498 | 1.13(0.79-1.61) |
| Peptococcus |  | 12 | ML | 0.18 | 0.14 | 0.197 | 1.19(0.91-1.56) |
| Peptococcus |  | 12 | cML-MA-BIC | 0.14 | 0.15 | 0.33 | 1.15(0.87-1.53) |
| Phascolarctobacterium |  | 9 | IVW | -0.17 | 0.31 | 0.587 | 0.84(0.46-1.56) |
| Phascolarctobacterium |  | 9 | MR-Egger | 2.07 | 1.33 | 0.162 | 7.96(0.59-107.1) |
| Phascolarctobacterium |  | 9 | WM | -0.22 | 0.32 | 0.501 | 0.81(0.43-1.51) |
| Phascolarctobacterium |  | 9 | ML | -0.19 | 0.24 | 0.431 | 0.83(0.52-1.32) |
| Phascolarctobacterium |  | 9 | cML-MA-BIC | -0.1 | 0.26 | 0.7 | 0.9(0.54-1.51) |
| Prevotella7 |  | 11 | IVW | -0.04 | 0.14 | 0.767 | 0.96(0.73-1.26) |
| Prevotella7 |  | 11 | MR-Egger | 1.31 | 0.73 | 0.105 | 3.69(0.89-15.28) |
| Prevotella7 |  | 11 | WM | 0.04 | 0.18 | 0.81 | 1.04(0.74-1.48) |
| Prevotella7 |  | 11 | ML | -0.04 | 0.12 | 0.732 | 0.96(0.75-1.22) |
| Prevotella7 |  | 11 | cML-MA-BIC | 0 | 0.14 | 0.98 | 1(0.77-1.31) |
| Prevotella9 |  | 15 | IVW | 0.08 | 0.19 | 0.677 | 1.08(0.75-1.57) |
| Prevotella9 |  | 15 | MR-Egger | -0.87 | 0.5 | 0.105 | 0.42(0.16-1.12) |
| Prevotella9 |  | 15 | WM | 0.42 | 0.23 | 0.061 | 1.53(0.98-2.39) |
| Prevotella9 |  | 15 | ML | 0.09 | 0.16 | 0.585 | 1.09(0.8-1.49) |
| Prevotella9 |  | 15 | cML-MA-BIC | 0.34 | 0.2 | 0.084 | 1.41(0.95-2.08) |
| RikenellaceaeRC9gutgroup |  | 11 | IVW | -0.02 | 0.13 | 0.899 | 0.98(0.76-1.27) |
| RikenellaceaeRC9gutgroup |  | 11 | MR-Egger | 1.08 | 0.78 | 0.2 | 2.94(0.64-13.53) |
| RikenellaceaeRC9gutgroup |  | 11 | WM | 0.13 | 0.17 | 0.443 | 1.14(0.82-1.57) |
| RikenellaceaeRC9gutgroup |  | 11 | ML | -0.02 | 0.12 | 0.886 | 0.98(0.78-1.24) |
| RikenellaceaeRC9gutgroup |  | 11 | cML-MA-BIC | 0.04 | 0.13 | 0.794 | 1.04(0.8-1.35) |
| Romboutsia |  | 13 | IVW | -0.41 | 0.22 | 0.067 | 0.67(0.43-1.03) |
| Romboutsia |  | 13 | MR-Egger | -1.35 | 0.6 | 0.046 | 0.26(0.08-0.84) |
| Romboutsia |  | 13 | WM | -0.17 | 0.3 | 0.571 | 0.84(0.47-1.52) |
| Romboutsia |  | 13 | ML | -0.42 | 0.21 | 0.05 | 0.66(0.43-1) |
| Romboutsia |  | 13 | cML-MA-BIC | -0.37 | 0.23 | 0.108 | 0.69(0.44-1.08) |
| Roseburia |  | 14 | IVW | -0.07 | 0.26 | 0.778 | 0.93(0.56-1.55) |
| Roseburia |  | 14 | MR-Egger | 0.12 | 0.83 | 0.885 | 1.13(0.22-5.76) |
| Roseburia |  | 14 | WM | -0.31 | 0.33 | 0.336 | 0.73(0.39-1.38) |
| Roseburia |  | 14 | ML | -0.07 | 0.23 | 0.763 | 0.93(0.59-1.48) |
| Roseburia |  | 14 | cML-MA-BIC | -0.2 | 0.25 | 0.434 | 0.82(0.5-1.34) |
| Ruminiclostridium5 |  | 11 | IVW | -0.11 | 0.25 | 0.66 | 0.89(0.54-1.47) |
| Ruminiclostridium5 |  | 11 | MR-Egger | -1.08 | 1.06 | 0.332 | 0.34(0.04-2.68) |
| Ruminiclostridium5 |  | 11 | WM | -0.19 | 0.35 | 0.593 | 0.83(0.42-1.64) |
| Ruminiclostridium5 |  | 11 | ML | -0.11 | 0.26 | 0.656 | 0.89(0.54-1.48) |
| Ruminiclostridium5 |  | 11 | cML-MA-BIC | -0.11 | 0.26 | 0.674 | 0.9(0.54-1.5) |
| Ruminiclostridium6 |  | 15 | IVW | 0.36 | 0.21 | 0.079 | 1.44(0.96-2.15) |
| Ruminiclostridium6 |  | 15 | MR-Egger | 0.09 | 0.52 | 0.869 | 1.09(0.39-3.05) |
| Ruminiclostridium6 |  | 15 | WM | 0.41 | 0.28 | 0.143 | 1.51(0.87-2.62) |
| Ruminiclostridium6 |  | 15 | ML | 0.38 | 0.2 | 0.059 | 1.46(0.99-2.15) |
| Ruminiclostridium6 |  | 15 | cML-MA-BIC | 0.33 | 0.22 | 0.125 | 1.39(0.91-2.12) |
| Ruminiclostridium9 |  | 9 | IVW | 0.35 | 0.29 | 0.234 | 1.42(0.8-2.51) |
| Ruminiclostridium9 |  | 9 | MR-Egger | 1.16 | 1.33 | 0.409 | 3.21(0.24-43.24) |
| Ruminiclostridium9 |  | 9 | WM | 0.27 | 0.38 | 0.479 | 1.31(0.62-2.75) |
| Ruminiclostridium9 |  | 9 | ML | 0.35 | 0.29 | 0.233 | 1.42(0.8-2.53) |
| Ruminiclostridium9 |  | 9 | cML-MA-BIC | 0.34 | 0.3 | 0.248 | 1.41(0.79-2.53) |
| RuminococcaceaeNK4A214group |  | 13 | IVW | -0.57 | 0.23 | 0.014 | 0.56(0.36-0.89) |
| RuminococcaceaeNK4A214group |  | 13 | MR-Egger | -1.08 | 0.78 | 0.191 | 0.34(0.07-1.55) |
| RuminococcaceaeNK4A214group |  | 13 | WM | -0.19 | 0.32 | 0.545 | 0.83(0.44-1.53) |
| RuminococcaceaeNK4A214group |  | 13 | ML | -0.59 | 0.22 | 0.007 | 0.55(0.36-0.85) |
| RuminococcaceaeNK4A214group |  | 13 | cML-MA-BIC | -0.51 | 0.26 | 0.047 | 0.6(0.36-0.99) |
| RuminococcaceaeUCG002 |  | 22 | IVW | -0.16 | 0.16 | 0.319 | 0.85(0.62-1.17) |
| RuminococcaceaeUCG002 |  | 22 | MR-Egger | -0.21 | 0.42 | 0.623 | 0.81(0.35-1.86) |
| RuminococcaceaeUCG002 |  | 22 | WM | -0.09 | 0.23 | 0.686 | 0.91(0.58-1.44) |
| RuminococcaceaeUCG002 |  | 22 | ML | -0.16 | 0.16 | 0.332 | 0.85(0.62-1.17) |
| RuminococcaceaeUCG002 |  | 22 | cML-MA-BIC | -0.17 | 0.16 | 0.302 | 0.84(0.61-1.17) |
| RuminococcaceaeUCG003 |  | 12 | IVW | 0.28 | 0.24 | 0.245 | 1.32(0.83-2.1) |
| RuminococcaceaeUCG003 |  | 12 | MR-Egger | 0.26 | 0.82 | 0.754 | 1.3(0.26-6.47) |
| RuminococcaceaeUCG003 |  | 12 | WM | 0.03 | 0.29 | 0.913 | 1.03(0.59-1.81) |
| RuminococcaceaeUCG003 |  | 12 | ML | 0.3 | 0.21 | 0.166 | 1.35(0.88-2.05) |
| RuminococcaceaeUCG003 |  | 12 | cML-MA-BIC | 0.21 | 0.24 | 0.376 | 1.24(0.77-1.97) |
| RuminococcaceaeUCG004 |  | 11 | IVW | 0.09 | 0.21 | 0.668 | 1.09(0.72-1.66) |
| RuminococcaceaeUCG004 |  | 11 | MR-Egger | 1.29 | 1.16 | 0.296 | 3.63(0.37-35.51) |
| RuminococcaceaeUCG004 |  | 11 | WM | 0.08 | 0.28 | 0.778 | 1.08(0.62-1.89) |
| RuminococcaceaeUCG004 |  | 11 | ML | 0.1 | 0.19 | 0.62 | 1.1(0.75-1.61) |
| RuminococcaceaeUCG004 |  | 11 | cML-MA-BIC | 0.07 | 0.2 | 0.728 | 1.07(0.72-1.59) |
| RuminococcaceaeUCG005 |  | 14 | IVW | 0.12 | 0.19 | 0.54 | 1.13(0.77-1.64) |
| RuminococcaceaeUCG005 |  | 14 | MR-Egger | 0.22 | 0.52 | 0.682 | 1.25(0.45-3.48) |
| RuminococcaceaeUCG005 |  | 14 | WM | 0.05 | 0.26 | 0.847 | 1.05(0.63-1.76) |
| RuminococcaceaeUCG005 |  | 14 | ML | 0.12 | 0.2 | 0.534 | 1.13(0.77-1.66) |
| RuminococcaceaeUCG005 |  | 14 | cML-MA-BIC | 0.1 | 0.2 | 0.602 | 1.11(0.75-1.65) |
| RuminococcaceaeUCG009 |  | 12 | IVW | -0.06 | 0.17 | 0.712 | 0.94(0.67-1.31) |
| RuminococcaceaeUCG009 |  | 12 | MR-Egger | -0.19 | 0.71 | 0.79 | 0.82(0.2-3.31) |
| RuminococcaceaeUCG009 |  | 12 | WM | -0.1 | 0.22 | 0.648 | 0.9(0.58-1.4) |
| RuminococcaceaeUCG009 |  | 12 | ML | -0.06 | 0.16 | 0.709 | 0.94(0.69-1.29) |
| RuminococcaceaeUCG009 |  | 12 | cML-MA-BIC | -0.14 | 0.18 | 0.444 | 0.87(0.62-1.23) |
| RuminococcaceaeUCG010 |  | 6 | IVW | 0.42 | 0.27 | 0.126 | 1.52(0.89-2.6) |
| RuminococcaceaeUCG010 |  | 6 | MR-Egger | 1.32 | 0.76 | 0.155 | 3.74(0.85-16.44) |
| RuminococcaceaeUCG010 |  | 6 | WM | 0.41 | 0.38 | 0.278 | 1.5(0.72-3.15) |
| RuminococcaceaeUCG010 |  | 6 | ML | 0.43 | 0.28 | 0.122 | 1.53(0.89-2.63) |
| RuminococcaceaeUCG010 |  | 6 | cML-MA-BIC | 0.4 | 0.29 | 0.162 | 1.5(0.85-2.63) |
| RuminococcaceaeUCG011 |  | 8 | IVW | -0.27 | 0.13 | 0.042 | 0.76(0.59-0.99) |
| RuminococcaceaeUCG011 |  | 8 | MR-Egger | -0.56 | 0.66 | 0.429 | 0.57(0.16-2.08) |
| RuminococcaceaeUCG011 |  | 8 | WM | -0.23 | 0.17 | 0.181 | 0.8(0.57-1.11) |
| RuminococcaceaeUCG011 |  | 8 | ML | -0.27 | 0.13 | 0.045 | 0.76(0.59-0.99) |
| RuminococcaceaeUCG011 |  | 8 | cML-MA-BIC | -0.27 | 0.14 | 0.048 | 0.77(0.59-1) |
| RuminococcaceaeUCG013 |  | 12 | IVW | 0.18 | 0.23 | 0.435 | 1.19(0.76-1.87) |
| RuminococcaceaeUCG013 |  | 12 | MR-Egger | -0.5 | 0.65 | 0.461 | 0.61(0.17-2.17) |
| RuminococcaceaeUCG013 |  | 12 | WM | 0.18 | 0.29 | 0.528 | 1.2(0.68-2.13) |
| RuminococcaceaeUCG013 |  | 12 | ML | 0.18 | 0.23 | 0.429 | 1.2(0.76-1.89) |
| RuminococcaceaeUCG013 |  | 12 | cML-MA-BIC | 0.2 | 0.24 | 0.402 | 1.22(0.77-1.94) |
| RuminococcaceaeUCG014 |  | 11 | IVW | 0.08 | 0.32 | 0.794 | 1.09(0.58-2.05) |
| RuminococcaceaeUCG014 |  | 11 | MR-Egger | 0.57 | 0.78 | 0.487 | 1.76(0.38-8.16) |
| RuminococcaceaeUCG014 |  | 11 | WM | 0.42 | 0.32 | 0.184 | 1.52(0.82-2.84) |
| RuminococcaceaeUCG014 |  | 11 | ML | 0.09 | 0.22 | 0.664 | 1.1(0.72-1.67) |
| RuminococcaceaeUCG014 |  | 11 | cML-MA-BIC | -0.13 | 0.35 | 0.712 | 0.88(0.45-1.73) |
| Ruminococcus1 |  | 10 | IVW | -0.12 | 0.23 | 0.607 | 0.89(0.56-1.4) |
| Ruminococcus1 |  | 10 | MR-Egger | -0.6 | 0.62 | 0.36 | 0.55(0.16-1.84) |
| Ruminococcus1 |  | 10 | WM | -0.13 | 0.31 | 0.678 | 0.88(0.48-1.61) |
| Ruminococcus1 |  | 10 | ML | -0.12 | 0.24 | 0.602 | 0.88(0.56-1.41) |
| Ruminococcus1 |  | 10 | cML-MA-BIC | -0.16 | 0.25 | 0.51 | 0.85(0.52-1.38) |
| Ruminococcus2 |  | 15 | IVW | 0.17 | 0.18 | 0.354 | 1.18(0.83-1.68) |
| Ruminococcus2 |  | 15 | MR-Egger | 0.56 | 0.43 | 0.219 | 1.75(0.75-4.08) |
| Ruminococcus2 |  | 15 | WM | 0.3 | 0.26 | 0.245 | 1.35(0.81-2.25) |
| Ruminococcus2 |  | 15 | ML | 0.17 | 0.18 | 0.342 | 1.19(0.83-1.7) |
| Ruminococcus2 |  | 15 | cML-MA-BIC | 0.22 | 0.19 | 0.253 | 1.25(0.85-1.82) |
| Ruminococcusgauvreauiigroup |  | 12 | IVW | -0.18 | 0.24 | 0.442 | 0.83(0.53-1.32) |
| Ruminococcusgauvreauiigroup |  | 12 | MR-Egger | 0.79 | 0.97 | 0.434 | 2.21(0.33-14.83) |
| Ruminococcusgauvreauiigroup |  | 12 | WM | -0.39 | 0.29 | 0.18 | 0.68(0.38-1.2) |
| Ruminococcusgauvreauiigroup |  | 12 | ML | -0.19 | 0.21 | 0.385 | 0.83(0.55-1.26) |
| Ruminococcusgauvreauiigroup |  | 12 | cML-MA-BIC | -0.25 | 0.23 | 0.27 | 0.78(0.49-1.22) |
| Ruminococcusgnavusgroup |  | 12 | IVW | 0.09 | 0.17 | 0.603 | 1.09(0.78-1.53) |
| Ruminococcusgnavusgroup |  | 12 | MR-Egger | 0.77 | 0.82 | 0.37 | 2.16(0.43-10.82) |
| Ruminococcusgnavusgroup |  | 12 | WM | 0.2 | 0.21 | 0.348 | 1.22(0.8-1.86) |
| Ruminococcusgnavusgroup |  | 12 | ML | 0.09 | 0.15 | 0.536 | 1.1(0.82-1.48) |
| Ruminococcusgnavusgroup |  | 12 | cML-MA-BIC | 0.07 | 0.16 | 0.657 | 1.07(0.78-1.47) |
| Ruminococcustorquesgroup |  | 9 | IVW | -0.09 | 0.3 | 0.773 | 0.92(0.51-1.65) |
| Ruminococcustorquesgroup |  | 9 | MR-Egger | 1.58 | 0.98 | 0.151 | 4.84(0.71-32.89) |
| Ruminococcustorquesgroup |  | 9 | WM | -0.28 | 0.39 | 0.478 | 0.76(0.35-1.64) |
| Ruminococcustorquesgroup |  | 9 | ML | -0.09 | 0.31 | 0.768 | 0.91(0.5-1.67) |
| Ruminococcustorquesgroup |  | 9 | cML-MA-BIC | -0.14 | 0.32 | 0.675 | 0.87(0.46-1.64) |
| Sellimonas |  | 9 | IVW | 0.04 | 0.12 | 0.743 | 1.04(0.83-1.31) |
| Sellimonas |  | 9 | MR-Egger | -0.32 | 0.68 | 0.65 | 0.72(0.19-2.76) |
| Sellimonas |  | 9 | WM | 0.02 | 0.15 | 0.921 | 1.02(0.75-1.37) |
| Sellimonas |  | 9 | ML | 0.04 | 0.12 | 0.737 | 1.04(0.83-1.31) |
| Sellimonas |  | 9 | cML-MA-BIC | 0.02 | 0.12 | 0.886 | 1.02(0.8-1.3) |
| Senegalimassilia |  | 5 | IVW | -0.16 | 0.28 | 0.573 | 0.85(0.49-1.48) |
| Senegalimassilia |  | 5 | MR-Egger | 0.78 | 1.11 | 0.535 | 2.17(0.25-19.12) |
| Senegalimassilia |  | 5 | WM | 0.03 | 0.34 | 0.937 | 1.03(0.53-2) |
| Senegalimassilia |  | 5 | ML | -0.16 | 0.27 | 0.536 | 0.85(0.5-1.43) |
| Senegalimassilia |  | 5 | cML-MA-BIC | -0.11 | 0.29 | 0.719 | 0.9(0.51-1.6) |
| Slackia |  | 6 | IVW | 0.2 | 0.29 | 0.499 | 1.22(0.69-2.16) |
| Slackia |  | 6 | MR-Egger | -0.25 | 2.12 | 0.91 | 0.78(0.01-49.15) |
| Slackia |  | 6 | WM | 0.09 | 0.29 | 0.767 | 1.09(0.62-1.92) |
| Slackia |  | 6 | ML | 0.21 | 0.22 | 0.337 | 1.24(0.8-1.92) |
| Slackia |  | 6 | cML-MA-BIC | 0.01 | 0.28 | 0.963 | 1.01(0.59-1.74) |
| Streptococcus |  | 13 | IVW | 0.04 | 0.23 | 0.851 | 1.04(0.67-1.64) |
| Streptococcus |  | 13 | MR-Egger | 0.66 | 0.8 | 0.426 | 1.94(0.4-9.3) |
| Streptococcus |  | 13 | WM | 0.01 | 0.3 | 0.973 | 1.01(0.56-1.83) |
| Streptococcus |  | 13 | ML | 0.05 | 0.23 | 0.843 | 1.05(0.66-1.66) |
| Streptococcus |  | 13 | cML-MA-BIC | 0.01 | 0.24 | 0.968 | 1.01(0.63-1.63) |
| Subdoligranulum |  | 11 | IVW | 0.38 | 0.28 | 0.175 | 1.47(0.84-2.56) |
| Subdoligranulum |  | 11 | MR-Egger | 0.88 | 0.75 | 0.274 | 2.4(0.55-10.53) |
| Subdoligranulum |  | 11 | WM | 0.52 | 0.32 | 0.11 | 1.68(0.89-3.16) |
| Subdoligranulum |  | 11 | ML | 0.41 | 0.24 | 0.089 | 1.5(0.94-2.4) |
| Subdoligranulum |  | 11 | cML-MA-BIC | 0.36 | 0.26 | 0.162 | 1.43(0.87-2.38) |
| Sutterella |  | 12 | IVW | 0.37 | 0.21 | 0.084 | 1.44(0.95-2.19) |
| Sutterella |  | 12 | MR-Egger | -0.04 | 0.91 | 0.963 | 0.96(0.16-5.75) |
| Sutterella |  | 12 | WM | 0.35 | 0.27 | 0.201 | 1.42(0.83-2.42) |
| Sutterella |  | 12 | ML | 0.37 | 0.21 | 0.083 | 1.45(0.95-2.21) |
| Sutterella |  | 12 | cML-MA-BIC | 0.37 | 0.22 | 0.088 | 1.45(0.95-2.21) |
| Terrisporobacter |  | 5 | IVW | -0.25 | 0.28 | 0.378 | 0.78(0.45-1.36) |
| Terrisporobacter |  | 5 | MR-Egger | -1.48 | 0.75 | 0.142 | 0.23(0.05-0.99) |
| Terrisporobacter |  | 5 | WM | -0.27 | 0.34 | 0.419 | 0.76(0.39-1.47) |
| Terrisporobacter |  | 5 | ML | -0.26 | 0.25 | 0.301 | 0.77(0.47-1.27) |
| Terrisporobacter |  | 5 | cML-MA-BIC | -0.24 | 0.27 | 0.373 | 0.79(0.47-1.33) |
| Turicibacter |  | 10 | IVW | -0.28 | 0.2 | 0.17 | 0.76(0.51-1.13) |
| Turicibacter |  | 10 | MR-Egger | -1.65 | 0.8 | 0.074 | 0.19(0.04-0.93) |
| Turicibacter |  | 10 | WM | -0.13 | 0.26 | 0.625 | 0.88(0.53-1.46) |
| Turicibacter |  | 10 | ML | -0.29 | 0.19 | 0.14 | 0.75(0.51-1.1) |
| Turicibacter |  | 10 | cML-MA-BIC | -0.22 | 0.22 | 0.307 | 0.8(0.52-1.22) |
| Tyzzerella3 |  | 13 | IVW | 0.05 | 0.13 | 0.694 | 1.05(0.81-1.37) |
| Tyzzerella3 |  | 13 | MR-Egger | -0.18 | 0.8 | 0.831 | 0.84(0.17-4.05) |
| Tyzzerella3 |  | 13 | WM | 0.05 | 0.19 | 0.773 | 1.05(0.73-1.52) |
| Tyzzerella3 |  | 13 | ML | 0.06 | 0.14 | 0.678 | 1.06(0.81-1.38) |
| Tyzzerella3 |  | 13 | cML-MA-BIC | 0.02 | 0.14 | 0.881 | 1.02(0.77-1.35) |
| Veillonella |  | 6 | IVW | -0.17 | 0.26 | 0.496 | 0.84(0.51-1.39) |
| Veillonella |  | 6 | MR-Egger | -1.16 | 2.06 | 0.602 | 0.31(0.01-17.6) |
| Veillonella |  | 6 | WM | -0.11 | 0.32 | 0.739 | 0.9(0.48-1.68) |
| Veillonella |  | 6 | ML | -0.18 | 0.26 | 0.493 | 0.84(0.5-1.39) |
| Veillonella |  | 6 | cML-MA-BIC | -0.17 | 0.26 | 0.517 | 0.84(0.5-1.41) |
| Victivallis |  | 10 | IVW | -0.06 | 0.12 | 0.629 | 0.94(0.74-1.2) |
| Victivallis |  | 10 | MR-Egger | -0.54 | 0.93 | 0.579 | 0.58(0.09-3.62) |
| Victivallis |  | 10 | WM | 0.02 | 0.15 | 0.913 | 1.02(0.75-1.37) |
| Victivallis |  | 10 | ML | -0.06 | 0.12 | 0.638 | 0.94(0.74-1.2) |
| Victivallis |  | 10 | cML-MA-BIC | -0.05 | 0.13 | 0.664 | 0.95(0.74-1.21) |

**Supplementary Table 2. All the available IVs details.**

| **Bacterial traits** | **SNP** | **Effect allele** | **Other allele** | **Gut microbiota** | | | | **male infertility** | | | | **Proxy SNP** | | **Target effect allele** | | **Target other allele** |
| --- | --- | --- | --- | --- | --- | --- | --- | --- | --- | --- | --- | --- | --- | --- | --- | --- |
|  |  |  |  | **Beta** | **SE** | | ***P* value** | **Beta** | **SE** | ***P* value** | |  |  |  |  |  |
| Allisonella | rs1901739 | T | G | 0.12 | 0.02 | | 3.59E-06 | -0.02 | 0.04 | 0.56 | | - | | - | | - |
|  | rs35110698 | C | T | 0.15 | 0.03 | | 5.72E-06 | 0.08 | 0.06 | 0.21 | | - | | - | | - |
|  | rs35778461 | C | T | 0.15 | 0.03 | | 1.21E-06 | 0.01 | 0.05 | 0.80 | | - | | - | | - |
|  | rs594561 | C | T | 0.11 | 0.03 | | 9.41E-06 | 0.01 | 0.04 | 0.79 | | - | | - | | - |
|  | rs602075 | A | G | 0.17 | 0.03 | | 3.57E-08 | 0.10 | 0.05 | 0.05 | | - | | - | | - |
|  | rs6742198 | G | A | 0.15 | 0.03 | | 3.35E-06 | 0.12 | 0.05 | 0.02 | | - | | - | | - |
|  | rs76904847 | G | A | 0.15 | 0.03 | | 6.09E-06 | 0.05 | 0.06 | 0.42 | | - | | - | | - |
|  | rs7898615 | T | G | 0.17 | 0.04 | | 8.87E-06 | -0.07 | 0.06 | 0.25 | | - | | - | | - |
| Anaerofilum | rs10794359 | C | T | 0.10 | 0.02 | | 2.23E-06 | 0.02 | 0.04 | 0.66 | | - | | - | | - |
|  | rs1563175 | A | C | 0.09 | 0.02 | | 5.54E-06 | -0.03 | 0.04 | 0.54 | | - | | - | | - |
|  | rs17012738 | T | G | 0.09 | 0.02 | | 7.24E-06 | 0.03 | 0.04 | 0.46 | | - | | - | | - |
|  | rs17096874 | T | C | 0.13 | 0.03 | | 2.86E-06 | -0.13 | 0.05 | 0.02 | | - | | - | | - |
|  | rs4244069 | A | G | 0.15 | 0.03 | | 9.81E-06 | -0.10 | 0.06 | 0.13 | | - | | - | | - |
|  | rs4506496 | G | A | 0.10 | 0.02 | | 1.49E-06 | -0.18 | 0.05 | 0.00 | | - | | - | | - |
|  | rs712981 | A | C | 0.10 | 0.02 | | 6.83E-07 | -0.03 | 0.04 | 0.50 | | - | | - | | - |
|  | rs79598899 | C | T | 0.18 | 0.04 | | 3.75E-07 | -0.04 | 0.10 | 0.73 | | - | | - | | - |
|  | rs816292 | C | T | 0.11 | 0.02 | | 2.64E-07 | -0.02 | 0.05 | 0.74 | | - | | - | | - |
|  | rs9299345 | C | T | 0.14 | 0.03 | | 8.04E-06 | 0.09 | 0.07 | 0.21 | | - | | - | | - |
| Anaerotruncus | rs10150232 | A | G | 0.06 | 0.01 | | 6.68E-06 | -0.03 | 0.05 | 0.61 | | - | | - | | - |
|  | rs11018566 | G | A | 0.16 | 0.04 | | 6.14E-06 | 0.04 | 0.09 | 0.70 | | - | | - | | - |
|  | rs115414803 | C | A | 0.14 | 0.03 | | 6.83E-06 | 0.29 | 0.09 | 0.00 | | - | | - | | - |
|  | rs1272208 | T | G | 0.06 | 0.01 | | 4.28E-06 | 0.01 | 0.05 | 0.78 | | - | | - | | - |
|  | rs1431492 | T | C | 0.07 | 0.01 | | 7.36E-06 | 0.03 | 0.06 | 0.63 | | - | | - | | - |
|  | rs17734739 | T | C | 0.07 | 0.01 | | 7.43E-06 | -0.01 | 0.06 | 0.85 | | - | | - | | - |
|  | rs34449434 | C | A | 0.05 | 0.01 | | 9.85E-06 | -0.03 | 0.04 | 0.48 | | - | | - | | - |
|  | rs4669806 | G | T | 0.06 | 0.01 | | 2.42E-06 | 0.08 | 0.05 | 0.11 | | - | | - | | - |
|  | rs6494922 | A | G | 0.09 | 0.02 | | 6.62E-06 | 0.12 | 0.10 | 0.21 | | - | | - | | - |
|  | rs6563550 | T | C | 0.09 | 0.02 | | 2.35E-07 | 0.01 | 0.08 | 0.89 | | - | | - | | - |
|  | rs7155595 | C | A | 0.05 | 0.01 | | 7.55E-06 | -0.03 | 0.05 | 0.51 | | - | | - | | - |
|  | rs8005030 | C | T | 0.06 | 0.01 | | 2.28E-06 | 0.11 | 0.05 | 0.02 | | - | | - | | - |
|  | rs9347879 | T | C | 0.05 | 0.01 | | 4.22E-06 | 0.10 | 0.04 | 0.02 | | - | | - | | - |
| Bacteroides | rs11585893 | G | A | 0.07 | 0.01 | | 1.80E-06 | -0.02 | 0.05 | 0.72 | | - | | - | | - |
|  | rs13207588 | G | A | 0.06 | 0.01 | | 7.49E-06 | -0.01 | 0.05 | 0.86 | | - | | - | | - |
|  | rs1340391 | C | T | 0.06 | 0.01 | | 6.73E-06 | -0.06 | 0.06 | 0.36 | | - | | - | | - |
|  | rs17619981 | T | G | 0.09 | 0.02 | | 2.69E-06 | -0.12 | 0.06 | 0.05 | | - | | - | | - |
|  | rs2023437 | C | T | 0.08 | 0.02 | | 5.02E-06 | -0.04 | 0.06 | 0.51 | | - | | - | | - |
|  | rs66474973 | G | T | 0.08 | 0.02 | | 6.81E-07 | 0.01 | 0.07 | 0.94 | | rs195028 | | G | | A |
|  | rs66710942 | C | T | 0.05 | 0.01 | | 5.86E-06 | -0.06 | 0.04 | 0.19 | | - | | - | | - |
|  | rs6795673 | C | T | 0.05 | 0.01 | | 3.38E-07 | -0.02 | 0.04 | 0.57 | | - | | - | | - |
|  | rs9507307 | C | T | 0.06 | 0.01 | | 2.13E-06 | 0.00 | 0.05 | 0.99 | | - | | - | | - |
| Barnesiella | rs11155559 | T | C | 0.10 | 0.02 | | 8.92E-06 | -0.19 | 0.07 | 0.01 | | - | | - | | - |
|  | rs113258194 | A | G | 0.10 | 0.02 | | 7.31E-06 | 0.17 | 0.07 | 0.02 | | rs115960966 | | T | | C |
|  | rs12909713 | T | C | 0.06 | 0.01 | | 4.95E-06 | 0.04 | 0.04 | 0.35 | | - | | - | | - |
|  | rs13242616 | C | T | 0.06 | 0.01 | | 2.29E-06 | 0.04 | 0.05 | 0.41 | | - | | - | | - |
|  | rs199035 | G | A | 0.06 | 0.01 | | 3.00E-06 | 0.00 | 0.04 | 1.00 | | - | | - | | - |
|  | rs2276875 | G | A | 0.07 | 0.01 | | 4.65E-07 | 0.10 | 0.05 | 0.05 | | - | | - | | - |
|  | rs2428166 | A | G | 0.17 | 0.03 | | 8.51E-07 | 0.26 | 0.19 | 0.19 | | - | | - | | - |
|  | rs35177866 | A | G | 0.09 | 0.02 | | 2.95E-06 | 0.03 | 0.08 | 0.72 | | - | | - | | - |
|  | rs60316894 | T | C | 0.12 | 0.03 | | 1.19E-06 | 0.07 | 0.08 | 0.36 | | rs61923473 | | A | | G |
|  | rs62251337 | G | A | 0.07 | 0.01 | | 4.24E-06 | 0.05 | 0.06 | 0.42 | | - | | - | | - |
|  | rs72684847 | C | T | 0.11 | 0.03 | | 6.76E-06 | -0.10 | 0.08 | 0.23 | | - | | - | | - |
|  | rs76181748 | T | C | 0.08 | 0.02 | | 6.78E-06 | 0.02 | 0.05 | 0.67 | | - | | - | | - |
|  | rs77455852 | G | T | 0.09 | 0.02 | | 3.16E-06 | -0.02 | 0.06 | 0.74 | | - | | - | | - |
|  | rs79795328 | G | A | 0.08 | 0.02 | | 4.23E-06 | 0.06 | 0.06 | 0.36 | | - | | - | | - |
| Intestinibacter | rs10805326 | G | A | 0.08 | 0.01 | | 3.55E-08 | 0.14 | 0.05 | 0.00 | | - | | - | | - |
|  | rs11109097 | C | T | 0.06 | 0.01 | | 5.49E-06 | 0.02 | 0.04 | 0.71 | | - | | - | | - |
|  | rs118030283 | A | G | 0.15 | 0.03 | | 2.67E-06 | -0.01 | 0.10 | 0.91 | | - | | - | | - |
|  | rs16938435 | C | T | 0.11 | 0.02 | | 1.80E-06 | 0.11 | 0.07 | 0.14 | | - | | - | | - |
|  | rs2098844 | T | C | 0.06 | 0.01 | | 6.79E-06 | 0.11 | 0.04 | 0.02 | | - | | - | | - |
|  | rs2702387 | A | G | 0.06 | 0.01 | | 4.26E-06 | -0.05 | 0.04 | 0.25 | | - | | - | | - |
|  | rs4327025 | A | G | 0.08 | 0.02 | | 1.64E-07 | -0.06 | 0.05 | 0.31 | | - | | - | | - |
|  | rs447950 | A | G | 0.06 | 0.01 | | 5.64E-06 | -0.02 | 0.04 | 0.70 | | - | | - | | - |
|  | rs478972 | C | T | 0.14 | 0.03 | | 1.82E-06 | 0.04 | 0.07 | 0.60 | | - | | - | | - |
|  | rs6062862 | A | G | 0.09 | 0.02 | | 6.68E-06 | 0.15 | 0.08 | 0.05 | | - | | - | | - |
|  | rs62430350 | T | C | 0.15 | 0.04 | | 6.84E-06 | 0.05 | 0.11 | 0.65 | | - | | - | | - |
|  | rs68093214 | C | T | 0.07 | 0.01 | | 9.26E-06 | -0.06 | 0.05 | 0.22 | | - | | - | | - |
|  | rs6875660 | C | T | 0.09 | 0.02 | | 3.06E-06 | -0.01 | 0.09 | 0.95 | | - | | - | | - |
|  | rs893394 | G | A | 0.06 | 0.01 | | 7.85E-06 | 0.02 | 0.04 | 0.57 | | - | | - | | - |
|  | rs9348442 | C | T | 0.10 | 0.02 | | 6.26E-06 | 0.11 | 0.06 | 0.08 | | - | | - | | - |
| Lactococcus | rs10417872 | T | G | 0.12 | 0.02 | | 1.29E-06 | 0.07 | 0.05 | 0.16 | | - | | - | | - |
|  | rs123059 | C | T | 0.14 | 0.03 | | 1.27E-06 | 0.00 | 0.05 | 0.94 | | - | | - | | - |
|  | rs12621813 | G | A | 0.11 | 0.02 | | 6.61E-06 | 0.11 | 0.05 | 0.02 | | - | | - | | - |
|  | rs17168302 | G | A | 0.19 | 0.04 | | 6.29E-06 | 0.09 | 0.07 | 0.19 | | - | | - | | - |
|  | rs2293361 | T | C | 0.20 | 0.04 | | 1.40E-06 | -0.03 | 0.09 | 0.72 | | - | | - | | - |
|  | rs4766997 | C | T | 0.11 | 0.02 | | 2.06E-06 | -0.05 | 0.04 | 0.26 | | - | | - | | - |
|  | rs55910161 | C | T | 0.15 | 0.03 | | 2.36E-06 | 0.06 | 0.07 | 0.39 | | - | | - | | - |
|  | rs6674304 | C | T | 0.20 | 0.04 | | 6.18E-06 | -0.01 | 0.11 | 0.93 | | - | | - | | - |
|  | rs7992246 | T | C | 0.10 | 0.02 | | 4.45E-06 | 0.05 | 0.04 | 0.29 | | rs932928 | | T | | C |
| Romboutsia | rs10279978 | G | A | 0.06 | 0.01 | | 1.17E-06 | 0.05 | 0.05 | 0.32 | | - | | - | | - |
|  | rs11221428 | C | T | 0.07 | 0.02 | | 6.49E-06 | -0.06 | 0.05 | 0.22 | | - | | - | | - |
|  | rs16843578 | T | C | 0.09 | 0.02 | | 5.08E-06 | -0.15 | 0.10 | 0.13 | | - | | - | | - |
|  | rs28603357 | C | T | 0.21 | 0.05 | | 8.52E-06 | -0.39 | 0.15 | 0.01 | | - | | - | | - |
|  | rs34302036 | A | G | 0.06 | 0.01 | | 5.88E-06 | -0.07 | 0.04 | 0.11 | | - | | - | | - |
|  | rs61841503 | G | A | 0.09 | 0.02 | | 4.00E-08 | 0.00 | 0.06 | 0.99 | | - | | - | | - |
|  | rs62504452 | G | A | 0.07 | 0.02 | | 4.66E-06 | 0.04 | 0.06 | 0.50 | | - | | - | | - |
|  | rs7109293 | A | G | 0.09 | 0.02 | | 6.98E-06 | -0.04 | 0.07 | 0.57 | | - | | - | | - |
|  | rs75200530 | G | T | 0.19 | 0.04 | | 5.07E-06 | -0.20 | 0.13 | 0.12 | | - | | - | | - |
|  | rs75987356 | A | G | 0.13 | 0.03 | | 6.71E-06 | -0.01 | 0.08 | 0.89 | | - | | - | | - |
|  | rs77702691 | G | A | 0.09 | 0.02 | | 7.37E-06 | 0.00 | 0.08 | 0.96 | | - | | - | | - |
|  | rs9389266 | T | G | 0.07 | 0.02 | | 9.38E-06 | -0.02 | 0.06 | 0.78 | | - | | - | | - |
|  | rs9567264 | C | T | 0.06 | 0.01 | | 5.76E-06 | 0.03 | 0.05 | 0.54 | | - | | - | | - |
| RuminococcaceaeNK4A214group | rs11241747 | C | T | 0.05 | 0.01 | | 6.59E-06 | 0.04 | 0.05 | 0.39 | | - | | - | | - |
|  | rs11586410 | A | G | 0.09 | 0.02 | | 3.66E-07 | 0.01 | 0.06 | 0.90 | | - | | - | | - |
|  | rs12642039 | C | T | 0.06 | 0.01 | | 3.43E-06 | -0.03 | 0.04 | 0.56 | | - | | - | | - |
|  | rs12731 | G | A | 0.05 | 0.01 | | 4.87E-06 | -0.09 | 0.04 | 0.04 | | - | | - | | - |
|  | rs13087692 | T | G | 0.06 | 0.01 | | 8.69E-06 | 0.00 | 0.05 | 0.98 | | - | | - | | - |
|  | rs136761 | A | G | 0.06 | 0.01 | | 8.15E-07 | -0.08 | 0.04 | 0.09 | | - | | - | | - |
|  | rs147475196 | G | A | 0.13 | 0.03 | | 4.72E-06 | -0.20 | 0.07 | 0.00 | | - | | - | | - |
|  | rs35559912 | C | T | 0.09 | 0.02 | | 4.89E-06 | 0.01 | 0.07 | 0.92 | | - | | - | | - |
|  | rs4814689 | T | C | 0.11 | 0.02 | | 4.55E-06 | 0.03 | 0.10 | 0.78 | | - | | - | | - |
|  | rs5994253 | G | A | 0.08 | 0.02 | | 2.35E-07 | -0.01 | 0.06 | 0.87 | | - | | - | | - |
|  | rs62027366 | T | C | 0.06 | 0.01 | | 6.58E-06 | -0.01 | 0.05 | 0.81 | | - | | - | | - |
|  | rs6681678 | T | C | 0.10 | 0.02 | | 9.05E-06 | -0.24 | 0.12 | 0.05 | | - | | - | | - |
|  | rs7573569 | T | C | 0.11 | 0.02 | | 3.23E-06 | -0.04 | 0.09 | 0.68 | | - | | - | | - |
| RuminococcaceaeUCG011 | rs10274562 | C | T | 0.11 | 0.02 | | 6.50E-06 | -0.02 | 0.04 | 0.57 | | - | | - | | - |
|  | rs12636310 | G | A | 0.13 | 0.03 | | 2.81E-06 | -0.07 | 0.05 | 0.13 | | - | | - | | - |
|  | rs12724320 | T | C | 0.12 | 0.02 | | 1.52E-06 | -0.03 | 0.04 | 0.47 | | - | | - | | - |
|  | rs1416041 | C | A | 0.18 | 0.03 | | 7.04E-08 | -0.08 | 0.05 | 0.14 | | - | | - | | - |
|  | rs2729556 | T | C | 0.11 | 0.02 | | 3.19E-06 | -0.02 | 0.04 | 0.66 | | - | | - | | - |
|  | rs4490371 | C | T | 0.11 | 0.02 | | 7.75E-06 | 0.00 | 0.04 | 0.94 | | - | | - | | - |
|  | rs79113084 | T | C | 0.15 | 0.03 | | 2.06E-06 | -0.02 | 0.07 | 0.81 | | - | | - | | - |
|  | rs9729514 | A | G | 0.18 | 0.04 | | 2.37E-06 | -0.03 | 0.07 | 0.71 | | - | | - | | - |
| **Supplementary Table 3. Horizontal pleiotropy of the association between gut microbiota and male infertility** | | | | | | | | | | | | | | |  |  |
| **Bacterial taxa (exposure)** | | | | | | **Egger_intercept** | | | | | **SE** | | **P-value** | |  |  |
| Allisonella | | | | | | -0.12 | | | | | 0.14 | | 0.444 | |  |  |
| Anaerofilum | | | | | | 0.05 | | | | | 0.13 | | 0.720 | |  |  |
| Anaerotruncus | | | | | | -0.04 | | | | | 0.06 | | 0.551 | |  |  |
| Bacteroides | | | | | | 0.00 | | | | | 0.09 | | 0.969 | |  |  |
| Barnesiella | | | | | | 0.04 | | | | | 0.08 | | 0.598 | |  |  |
| Intestinibacter | | | | | | -0.02 | | | | | 0.06 | | 0.769 | |  |  |
| Lactococcus | | | | | | 0.05 | | | | | 0.09 | | 0.574 | |  |  |
| Romboutsia | | | | | | 0.08 | | | | | 0.05 | | 0.122 | |  |  |
| Ruminococcaceae(NK4A214group) | | | | | | 0.04 | | | | | 0.06 | | 0.505 | |  |  |
| Ruminococcaceae(UCG011) | | | | | | 0.04 | | | | | 0.09 | | 0.667 | |  |  |

| **Supplementary Table 4. MRPRESSO of the association between gut microbiota and male infertility.** | | | | | | | | |
| --- | --- | --- | --- | --- | --- | --- | --- | --- |
| **Bacterial taxa (exposure)** | **MR Analysis** | **Causal Estimate** | **SD** | **T** | **P-value** | **RSSobs** | **Global test P-value** | **Remove SNP** |
| Allisonella | MR-PRESSO | 0.25 | 0.15 | 1.69 | 0.135 | 12.67 | 0.246 | - |
| Anaerofilum | MR-PRESSO | -0.35 | 0.21 | -1.66 | 0.131 | 23.95 | 0.027 | rs4506496 |
| Anaerofilum | Outlier-corrected | -0.20 | 0.17 | -1.21 | 0.259 |  |  |  |
| Anaerotruncus | MR-PRESSO | 0.67 | 0.28 | 2.41 | 0.033 | 21.24 | 0.125 | - |
| Bacteroides | MR-PRESSO | -0.54 | 0.18 | -3.06 | 0.016 | 4.37 | 0.902 | - |
| Barnesiella | MR-PRESSO | 0.34 | 0.25 | 1.36 | 0.198 | 23.51 | 0.105 | - |
| Intestinibacter | MR-PRESSO | 0.42 | 0.23 | 1.79 | 0.096 | 27.30 | 0.066 | - |
| Lactococcus | MR-PRESSO | 0.25 | 0.15 | 1.72 | 0.124 | 11.33 | 0.372 | - |
| Romboutsia | MR-PRESSO | -0.41 | 0.22 | -1.83 | 0.092 | 15.92 | 0.347 | - |
| Ruminococcaceae(NK4A214group) | MR-PRESSO | -0.57 | 0.23 | -2.45 | 0.031 | 17.65 | 0.286 | - |
| Ruminococcaceae(UCG011) | MR-PRESSO | -0.27 | 0.06 | -4.26 | 0.004 | 2.13 | 0.980 | - |

| **Supplementary Table 5. MR analysis again by removing the outliers** | | | | | |  |
| --- | --- | --- | --- | --- | --- | --- |
| **Bacterial taxa (exposure)** | **Nsnp** | **Methods** | **Beta** | **SE** | **P value** | **OR（95% CI）** |
| Anaerofilum | 9 | IVW | -0.20 | 0.17 | 0.225 | 0.82(0.59-1.13) |
| Anaerofilum | 9 | MR-Egger | -1.02 | 0.88 | 0.282 | 0.36(0.06-2.01) |
| Anaerofilum | 9 | WM | -0.21 | 0.23 | 0.356 | 0.81(0.52-1.26) |
| Anaerofilum | 9 | ML | -0.20 | 0.16 | 0.193 | 0.82(0.60-1.11) |
| Anaerofilum | 9 | cML-MA-BIC | -0.17 | 0.17 | 0.329 | 0.84(0.60-1.19) |

| **Supplementary Table 6. Heterogeneity of gut microbiota instrumental variables.** | | | | | | |
| --- | --- | --- | --- | --- | --- | --- |
| **Bacterial taxa (exposure)** | **Rucker's Q** | **df** | **P-value** | **Cochran's Q** | **df** | **P-value** |
| Allisonella | 9.54 | 7 | 0.216 | 8.58 | 6 | 0.198 |
| Anaerofilum | 19.17 | 8 | 0.014 | 19.50 | 9 | 0.021 |
| Anaerotruncus | 17.16 | 11 | 0.103 | 17.75 | 12 | 0.123 |
| Bacteroides | 3.35 | 7 | 0.851 | 3.35 | 8 | 0.910 |
| Barnesiella | 19.76 | 12 | 0.072 | 20.25 | 13 | 0.089 |
| Intestinibacter | 23.55 | 13 | 0.036 | 23.71 | 14 | 0.050 |
| Lactococcus | 8.39 | 7 | 0.299 | 8.81 | 8 | 0.358 |
| Romboutsia | 10.83 | 11 | 0.458 | 13.63 | 12 | 0.325 |
| Ruminococcaceae(NK4A214group) | 13.82 | 11 | 0.243 | 14.42 | 12 | 0.275 |
| Ruminococcaceae(UCG011) | 1.38 | 6 | 0.967 | 1.59 | 7 | 0.979 |
